# Cardiac-Specific SOCS3 Deletion Prevents *In Vivo* Myocardial Ischemia Reperfusion Injury through Sustained Activation of Cardioprotective Signaling Molecules

**DOI:** 10.1371/journal.pone.0127942

**Published:** 2015-05-26

**Authors:** Takanobu Nagata, Hideo Yasukawa, Sachiko Kyogoku, Toyoharu Oba, Jinya Takahashi, Shoichiro Nohara, Tomoko Minami, Kazutoshi Mawatari, Yusuke Sugi, Koutatsu Shimozono, Sylvain Pradervand, Masahiko Hoshijima, Hiroki Aoki, Yoshihiro Fukumoto, Tsutomu Imaizumi

**Affiliations:** 1 Division of Cardiovascular Medicine, Department of Internal Medicine, Kurume University School of Medicine, Kurume, Fukuoka, Japan; 2 Cardiovascular Research Institute, Kurume University, Kurume, Fukuoka, Japan; 3 Lausanne Genomic Technologies Facility (LGTF), University of Lausanne, Lausanne, Switzerland; 4 Department of Medicine, University of California San Diego, La Jolla, California, United States of America; 5 Fukuoka Sanno Hospital and International University of Health and Welfare, Fukuoka, Japan; University of Pittsburgh, UNITED STATES

## Abstract

Myocardial ischemia reperfusion injury (IRI) adversely affects cardiac performance and the prognosis of patients with acute myocardial infarction. Although myocardial signal transducer and activator of transcription (STAT) 3 is potently cardioprotective during IRI, the inhibitory mechanism responsible for its activation is largely unknown. The present study aimed to investigate the role of the myocardial suppressor of cytokine signaling (SOCS)-3, an intrinsic negative feedback regulator of the Janus kinase (JAK)-STAT signaling pathway, in the development of myocardial IRI. Myocardial IRI was induced in mice by ligating the left anterior descending coronary artery for 1 h, followed by different reperfusion times. One hour after reperfusion, the rapid expression of JAK-STAT–activating cytokines was observed. We precisely evaluated the phosphorylation of cardioprotective signaling molecules and the expression of SOCS3 during IRI and then induced myocardial IRI in wild-type and cardiac-specific SOCS3 knockout mice (SOCS3-CKO). The activation of STAT3, AKT, and ERK1/2 rapidly peaked and promptly decreased during IRI. This decrease correlated with the induction of SOCS3 expression up to 24 h after IRI in wild-type mice. The infarct size 24 h after reperfusion was significantly reduced in SOCS3-CKO compared with wild-type mice. In SOCS3-CKO mice, STAT3, AKT, and ERK1/2 phosphorylation was sustained, myocardial apoptosis was prevented, and the expression of anti-apoptotic Bcl-2 family member myeloid cell leukemia-1 (Mcl-1) was augmented. Cardiac-specific SOCS3 deletion led to the sustained activation of cardioprotective signaling molecules including and prevented myocardial apoptosis and injury during IRI. Our findings suggest that SOCS3 may represent a key factor that exacerbates the development of myocardial IRI.

## Introduction

For patients with acute myocardial infarction, an early reperfusion of the occluded coronary artery using primary percutaneous coronary intervention is the best way for limiting infarct size, which contributes to preserving left ventricular (LV) contraction and preventing the onset of heart failure [1, 2]. Although reperfusion can salvage the myocardium, reperfusion itself paradoxically induces further cardiomyocyte death, which is generally known as myocardial ischemia reperfusion injury (IRI) [3–5]. Numerous strategies ameliorate myocardial IRI in animals. Yet, the translation of these beneficial effects to the clinical setting has been disappointing [4, 5]. Therefore, it will be beneficial to determine the mechanisms underlying myocardial IRI and identify novel therapeutic targets that will effectively suppress this adverse process.

Myocardial IRI induces intracellular calcium overload, oxidative stress, and inflammation, which in turn initiate myocardial cell death, including apoptosis [3–5]. Apoptosis is a main underlying mechanism of myocardial IRI and can be reversed by the activation of pro-survival signaling pathways [6–8]. Animal studies provide many lines of evidence suggesting that myocardial Janus kinase (JAK)/signal transducer and activator of transcription (STAT) 3 pathway is a potent pro-survival signaling pathway during IRI [9–15]. Hearts from mice with a cardiac-specific deletion of STAT3 during IRI have larger infarct size with increased numbers of apoptotic cardiomyocytes and show greatly increased mortality [16]. STAT3 has been implicated in the cardioprotection conferred by different types of preconditioning (ischemic and pharmacological) and postconditioning [17–24]. Recently, STAT3 has been identified in the cardiomyocyte mitochondria, where it modulates mitochondrial respiration, regulates mitochondria-mediated apoptosis, and inhibits the opening of mitochondrial permeability transition pores [25, 26]. Thus, myocardial STAT3 is centrally involved in cardioprotection during IRI [27]. Although the activation of STAT3 occurs transiently during myocardial IRI, the mechanism of myocardial STAT3 inactivation during IRI is largely unknown.

The JAK-STAT pathway can be negatively regulated at several steps through distinct mechanisms [28, 29]. The suppressor of cytokine signaling (SOCS) family of proteins are major specific regulators of the activation of the JAK-STAT pathway [29, 30]. We and other groups have identified SOCS proteins as cytokine-inducible intrinsic inhibitors of JAK-STAT signaling pathways [29, 31]. Among the members of this family, SOCS1 and SOCS3 potently suppress cytokine activity by interacting with JAK as a pseudosubstrate and inhibiting its kinase activity [32]. The expression of SOCS3 is induced by JAK-STAT–activating cytokines and myocardial insults such as viral infection or pressure overload [33–35]. We previously reported that the forced expression of SOCS3 inhibits cytokine-promoted cardiomyocyte survival *in vitro* and cardiac-specific transgenic expression of SOCS3 facilitates coxsackievirus-induced cardiac injury in mice by inhibiting the activation of multiple signaling molecules downstream of JAK [33–35]. In contrast, the deletion of myocardial SOCS3 in mice enhances the activation of multiple JAK-STAT signaling pathways and prevents cardiac injury and remodeling after permanent coronary artery ligation [36]. Thus, the expression of SOCS3 is induced by cytokines or myocardial insults and is centrally involved in the progression of myocardial injury. Therefore, we hypothesized that myocardial IRI induces SOCS3 expression in the mouse heart and that the deletion of myocardial SOCS3 would prevent IRI in mice. To test this hypothesis, we precisely determined the activation of JAK-STAT signaling pathways and expression of SOCS3 during myocardial IRI and compared induced myocardial IRI in wild-type (WT) mice versus cardiac-specific SOCS3 knockout mice (SOCS3-CKO).

In this study, we showed that the activation of STAT3 rapidly peaked but was promptly suppressed after IRI, which correlated with the induction of the expression of SOCS3 for as long as 24 h after IRI in WT mice. The cardiac-specific deletion of SOCS3 induced the sustained activation of cardioprotective signaling molecules including STAT3 and inhibited myocardial apoptosis through the increased expression of anti-apoptotic Bcl-2 family member myeloid cell leukemia-1 (Mcl-1), resulting in the prevention of myocardial IRI.

## Materials and Methods

### Generation of cardiac-specific SOCS3 knockout mice

We generated SOCS3^flox/flox^ mice, which carried a SOCS3 allele flanked by loxP sites, as reported previously [37]. To delete SOCS3 in the myocardium, SOCS3^flox/flox^ mice were bred with mice harboring a transgene encoding Cre recombinase driven by the α-myosin heavy chain (α-MHC)-promoter [38]. All SOCS3^flox/flox^ (WT) and SOCS3^flox/flox^/α-MHC-Cre (SOCS3-CKO) mice used in this study were males (8- to 10-week-old) in a Balb/c background. SOCS3-CKO pups were born in an expected Mendelian ratio and grew normally to adulthood. This study was carried out in strict accordance with the recommendations in the Guide for the Care and Use of Laboratory Animals of the National Institutes of Health. The study protocol was approved by the Institutional Animal Care and Use Committee of Kurume University School of Medicine (Permit Number: 2014-095-1). All procedures on the mice were performed under general anesthesia with isoflurane (5% in 100% oxygen for induction; 1–2% in 100% oxygen for maintenance) using an animal anesthesia machine (model TK-5, Bio Machinery, Chiba, Japan), and all efforts were made to minimize suffering.

### *In vivo* mouse model of myocardial ischemia reperfusion injury

Animals were anesthetized with inhaled isoflurane administered using an endotracheal tube, and positive pressure ventilation was provided with a constant-volume ventilator operating on the Starling principle (HSE MiniVent, Harvard Apparatus GmbH). After the thoracic cavity was opened by left thoracotomy, an 8–0 prolene suture was passed under the left anterior descending (LAD) coronary artery at the inferior edge of the left atrium and tied to produce an occlusion [39]. Ischemia was confirmed by blanching downstream of the ligation and through persistent ST segment elevation on the electrocardiogram. Body temperature was maintained at 37°C using a heating pad, and temperature was monitored using a rectal thermometer. After 60 min of ischemia, the ligature was released to reperfuse the LAD coronary artery. Reperfusion was confirmed by the visible restoration of color in the ischemic myocardium and inversion of the T wave on the electrocardiogram [39]. The chest was closed with continuous 6–0 prolene sutures. The endotracheal tube was removed after spontaneous respiration resumed. A sham operation included all procedures, excepting the ligation of the LAD coronary artery.

### Evans blue dye and triphenyltetrazolium chloride staining

Twenty-four hours after reperfusion, each mouse was anesthetized as described above, and the chest was reopened. The LAD coronary artery was re-occluded, and the heart was perfused with 5% Evans blue dye that indicated the normally perfused area, while the absence of staining indicated the ischemic area [i.e., area at risk (AAR)]. The heart was excised, and the LV was cut into five transverse slices from the apex to the base. The slices were incubated in 1% triphenyltetrazolium chloride (TTC) solution at 37°C for 10 min, photographed with a digital camera (Nikon, DXM1200, Japan), and weighed. For each image, the area lacking TTC staining (i.e., infarct area) and the AAR and LV areas were measured with a planimeter using Image-Pro PLUS software (version 621.490). For each slice, the ratios of infarct to LV area and AAR to LV area were determined and multiplied by the slice weight to calculate net infarct area and AAR weights, respectively. The values for each slice were then summed over all slices. The total infarct area weight was divided by the total AAR weight (infarct area/AAR) to obtain the infarct size, and the total AAR weight was divided by the total LV weight (AAR/LV) to obtain the ischemic size [36, 39].

### Bio-Plex analysis

After centrifugation, serum samples were frozen and stored at −80°C until use. Serum levels of granulocyte colony-stimulating factor (G-CSF), interleukin (IL)-6, granulocyte macrophage CSF (GM-CSF), and leukemia inhibitory factor (LIF) pre-ischemia and 1 h after IRI were measured on a Bio-Plex system (Bio-Rad Laboratories).

### Western blot analysis

At the indicated time after reperfusion, whole LV tissues were collected and homogenized in lysis buffer containing 25 mM Hepes (pH 7.5), 1% Triton X100, 150 mM NaCl, 10% glycerol, 1 mM sodium orthovanadate, 50 mM NaF, 10 mM sodium pyrophosphate, and protease inhibitor cocktail (Sigma Chemical Co.). The total cell extracts were resolved by SDS-PAGE, and western blot analysis was performed as described previously [33, 34]. Antibodies against tyrosine-phosphorylated STAT3 (pY-STAT3; #9145, D3A7, rabbit monoclonal, 1:200 dilution), serine-phosphorylated STAT3 (pS-STAT3; #9134, rabbit polyclonal, 1:200 dilution), STAT3 (#9132, rabbit polyclonal, 1:200 dilution), phosphorylated AKT (p-AKT; #4060, D9E, rabbit monoclonal, 1:200 dilution), AKT (#9272, rabbit polyclonal, 1:200 dilution), phosphorylated ERK1/2 (p-ERK1/2; #4370, D13.14.4E, rabbit monoclonal, 1:200 dilution), ERK1/2 (#9102, rabbit polyclonal, 1:200 dilution), cleaved caspase-3 (#9664, 5A1E, rabbit monoclonal, 1:200 dilution), cleaved caspase-8 (#8592, D5B2, rabbit monoclonal, 1:200 dilution), Bcl-xL (#2764, 54H6, rabbit monoclonal, 1:200 dilution), Bad (#9292, rabbit polyclonal, 1:200 dilution), Bax (#2772, rabbit polyclonal, 1:200 dilution), and Mcl-1 (#5453, D35A5, rabbit monoclonal, 1:200 dilution) were purchased from Cell Signaling Technology.

### SOCS3 immunoprecipitation

SOCS3 immunoprecipitation was performed as previously described [33, 37]. In brief, at the indicated time after reperfusion, whole LV tissues were collected, and immunoprecipitation was conducted using a Dynabeads Protein A Immunoprecipitation Kit (#10006D, Life Technologies) according to manufacturer’s protocol. Anti-SOCS3 rabbit polyclonal antibodies (#18391, C204 and #18395, C005) for immunoprecipitation were purchased from Immuno-Biological Laboratory, and anti-SOCS3 goat polyclonal antibodies (sc-7009, M20 and sc-7010, S19, 1:200 dilution) for western blotting were purchased from Santa Cruz Biotechnology.

### Isolation of mitochondrial and cytosolic fractions

Six hours after reperfusion, whole LV tissues were collected and homogenized using a glass tissue grinder. The homogenate was centrifuged at 1,000 × *g* for 3 min. The pellet was suspended, and the mitochondrial and cytosolic fractions were isolated using a Mitochondria Isolation Kit (#89801, Thermo Scientific), according to manufacturer’s protocol. All steps were performed at 4°C. Western blot analysis was performed using antibodies against cytochrome c (#556433, 7H8.2C12, mouse monoclonal, 1:200 dilution, BD Pharmingen), the cytosolic marker α-Tubulin (#sc-8035, TU-02, mouse monoclonal, 1:200 dilution, Santa Cruz Biotechnology), and the mitochondrial marker VDAC (#4661, D73D12, mouse monoclonal, 1:200 dilution, Cell Signaling Technology).

### TUNEL assay

To detect apoptosis, a terminal deoxynucleotidyl transferase dUTP nick end labeling (TUNEL) assay was performed using an *In Situ* Apoptosis Detection kit (Takara), according to the manufacturer’s protocol [33, 34]. Images were acquired at 400× magnification, and 25 random, high-power fields from each heart sample were selected and quantified.

### Immunohistochemical staining

Freshly isolated hearts were fixed in 4% paraformaldehyde (PFA), dehydrated, embedded in paraffin and sectioned. Sections were subjected to immunohistochemical staining using a rabbit monoclonal antibody raised against pY-STAT3 (#9145, D3A7, 1:200 dilution, Cell Signaling Technology) [36].

### RNA extraction and real-time PCR

The total LV RNA was isolated using TRIzol (Invitrogen), as previously described [33, 34], and 1 μg of total RNA was converted into cDNA. The expression profiles of common cytokines were obtained using the RT2 Profiler PCR array for murine common cytokines (SABioscience) according to the manufacturer’s instructions. PCR was performed using the StepOne real-time PCR system (Applied Biosystems). The ΔΔC_t_ method was used to analyze the expression level of each gene. After PCR, the dissociation curve for each gene was evaluated to exclude genes with nonspecific amplification or undetectable expression. The expression profile of each gene was displayed as a heat map created using MeV MultiExperiment Viewer 4.1 [36]. We also performed real-time PCR assays to assess the gene expression of mouse SOCS3, Mcl1, Bcl2, and GAPDH using the corresponding primer pairs (Applied Biosystems, #Mm00545913_s1, #Mm00725832_s1, Mm00477631_m1, and #Mm99999915_g1, respectively) using the StepOnePlus Real-Time PCR System (Applied Biosystems) [36].

### Echocardiography

Transthoracic echocardiographic studies were performed on mice administered light anesthesia using a Vevo770 ultrasound machine (VisualSonics Inc) equipped with a 30-MHz probe. Mice were anesthetized with isoflurane and subjected to echocardiography, as previously described [33, 34, 36].

### Statistical analysis

Data are expressed as the mean ± standard error of the mean (SE). Multiple group comparisons were performed using one-way analysis of variance (ANOVA) followed by the Bonferroni procedure for comparison of means. Comparisons between two groups were analyzed using a two-tailed Student’s *t*-test or two-way ANOVA. A p value of < 0.05 was considered to indicate statistical significance.

## Results

### Rapid inductions of JAK-STAT–activating cytokines during myocardial IRI

**Fig 1:**
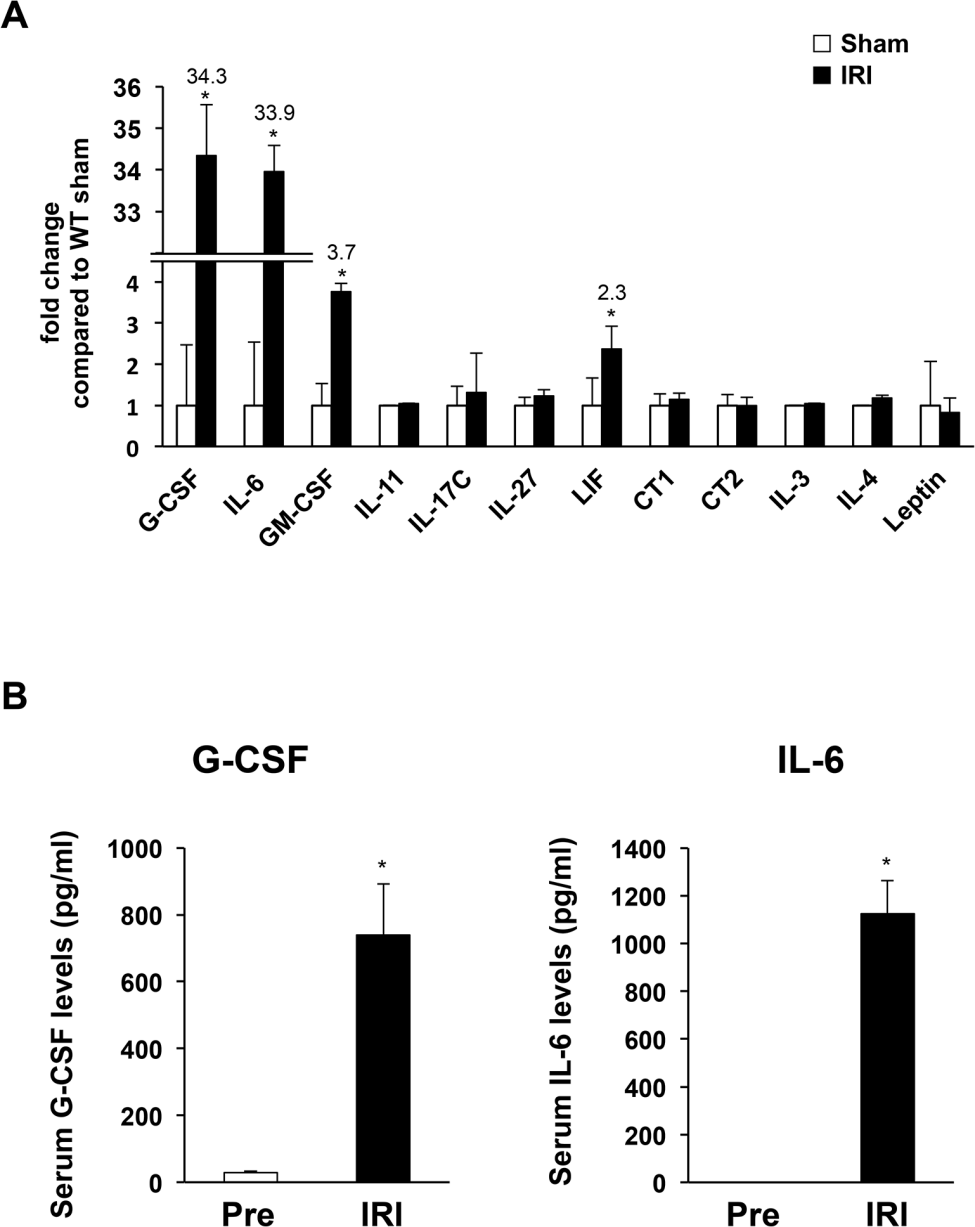
Expression of JAK-STAT–activating cytokines in wild-type (WT) mice during myocardial IRI. (**A**) mRNA was prepared from the left ventricle (LV) of WT mice 1 h after reperfusion, and real-time PCR analyses for cytokines were performed. Values normalized to GAPDH are expressed as fold change from the values of sham mice (n = 5 per group). (**B**) Serum samples were collected from WT mice at 1 h after reperfusion, and we performed Bio-Plex analysis for cytokines (n = 5 per group). *P < 0.05 vs. pre-ischemia.

First, we examined the myocardial expression of common cytokines and growth factors 1 h after reperfusion in WT mice. Using quantitative real-time PCR array containing primers for 168 genes coding for murine common cytokines and growth factors, we detected upregulation of four genes encoding cytokines that activate the JAK-STAT signaling pathway, including G-CSF, IL-6, and LIF (**Fig 1A**). Serum levels of G-CSF and IL-6 were markedly increased at 1 h after IRI (**Fig 1B**). In contrast, circulating GM-CSF and LIF were undetectable level at 1 h after IRI (data not shown).

### Rapid suppression of the phosphorylation of cardioprotective signaling molecules during myocardial IRI

**Fig 2:**
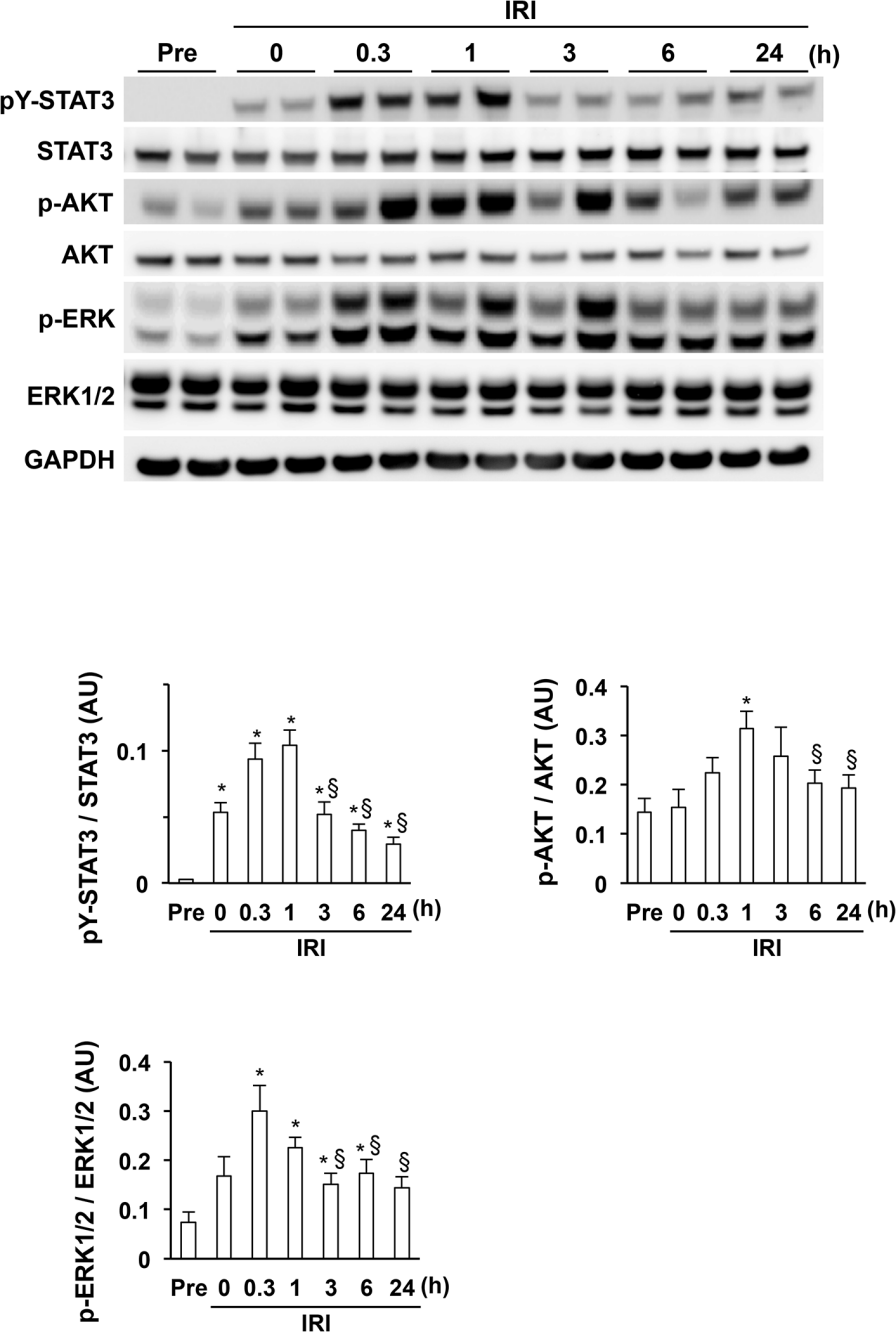
Activation of cardioprotective signaling molecules in wild-type (WT) mice during myocardial IRI. Total cell lysates were prepared from the left ventricle of WT mice at the indicated times after reperfusion. Blots were probed using antibodies against tyrosine-phosphorylated STAT3 (pY-STAT3), STAT3, phosphorylated AKT (p-AKT), AKT, phosphorylated ERK1/2 (p-ERK1/2), and GAPDH. The graphs represent quantitative differences in expression between the ratios of pY-STAT3 to STAT, p-AKT to AKT, and p-ERK1/2 to ERK1/2 (n = 6 per group). *P < 0.05 vs. pre-ischemia, §*P* < 0.05 vs. 1 h (pY-STAT3 and p-AKT) or 0.3 h (p-ERK1/2) after reperfusion. AU, arbitrary units; GAPDH, glyceraldehyde 3-phosphate dehydrogenase; IRI, ischemia reperfusion injury.

At pre-ischemia and during IRI, we performed western blot analysis to precisely determine the phosphorylation of cardioprotective signaling molecules downstream of JAK in WT mice. STAT3 phosphorylation was undetectable pre-ischemia, faint at 0 h, peaked 1 h after reperfusion, and then decreased significantly at 3 h after reperfusion (**Fig 2**). AKT phosphorylation was faint pre-ischemia, increased at 0 h, peaked 1 h after reperfusion, and then decreased significantly at 6 h after reperfusion (**Fig 2**). ERK1/2 phosphorylation was faint pre-ischemia, increased at 0 h, peaked 0.3 h after reperfusion, and then decreased significantly at 3 h after reperfusion (**Fig 2**). Thus, the IRI-induced activation of cardioprotective signaling molecules decreased rapidly after reperfusion, suggesting a mechanism that inhibits the activities of cardioprotective signaling molecules during IRI.

### Confirmation of myocardial-specific SOCS3 deletion in SOCS3-CKO mice

**Fig 3:**
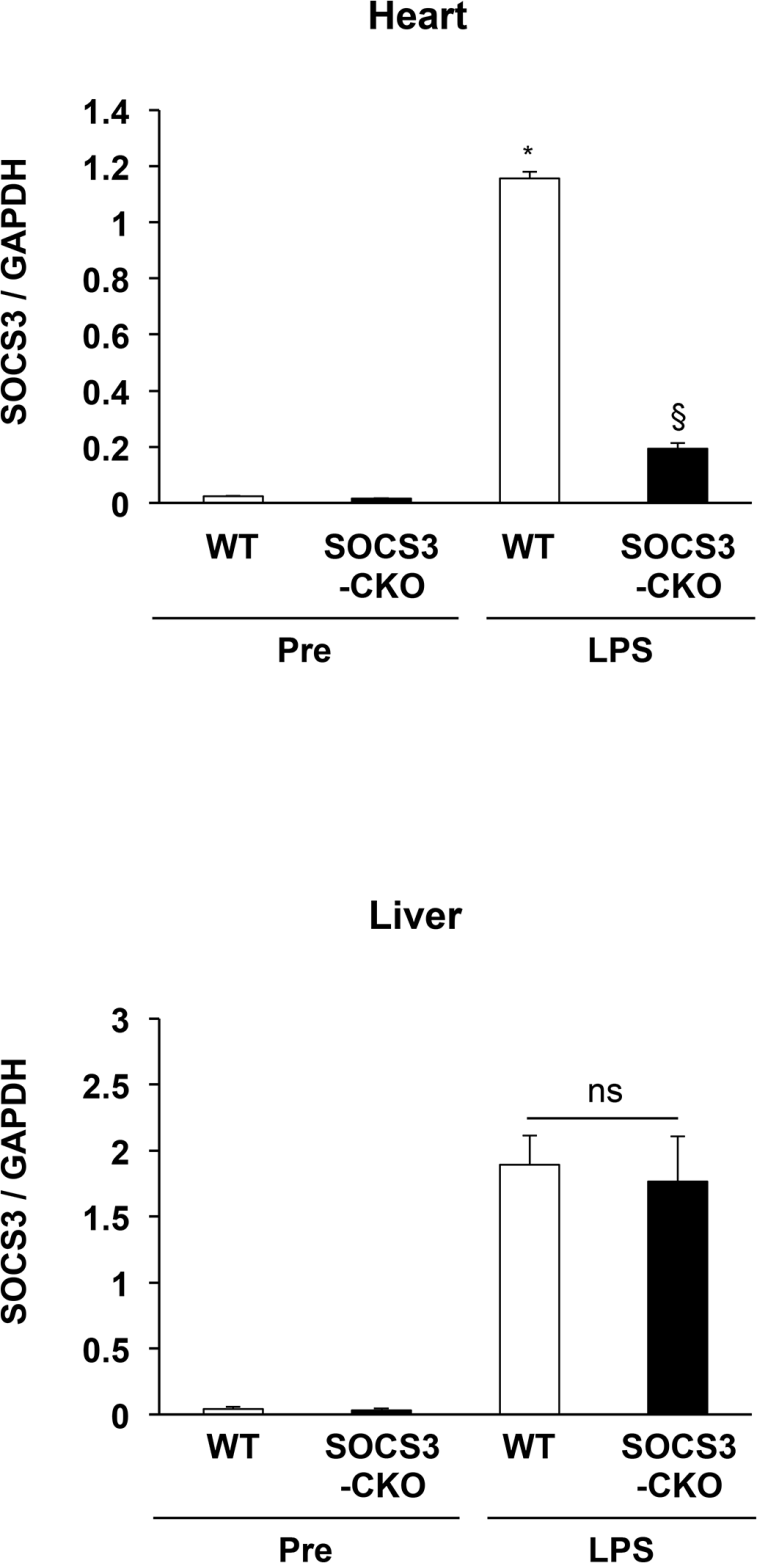
Confirmation of myocardial-specific SOCS3 deletion in SOCS3-CKO mice. mRNA was prepared from the hearts and livers of wild-type (WT) mice 3 h after lipopolysaccharide (LPS) injection, and real-time PCR analyses for SOCS3 were performed. Values normalized to GAPDH are expressed (n = 5 per group). *P < 0.05 vs. WT pre-injection, §*P* < 0.05 vs. WT LPS injection.

For systemic induction of SOCS3 expression, we intraperitoneally injected lipopolysaccharide (LPS, 20 mg/kg) into mice and performed real-time PCR of SOCS3 in the heart and liver. We confirmed that SOCS3 mRNA expression was induced in LPS-injected hearts from WT mice, and was markedly reduced in LPS-injected hearts from SOCS3-CKO mice (**Fig 3**). In contrast, the upregulation of SOCS3 expression after LPS injection was comparable between WT and SOCS3-CKO liver (**Fig 3**). Thus, the deletion of SOCS3 via the α-MHC-promoter Cre recombinase is specific to the myocardium. Echocardiography revealed no difference in left ventricular function or wall thickness in SOCS3-CKO mice when compared with WT mice (**S1 Fig**). Hematoxylin/eosin and Mallory-Azan staining of hearts from 10-week-old SOCS3-CKO mice revealed no evidence of necrosis, cardiac fibrosis, or hypertrophy (**S2 Fig**). Thus, histological examination and echocardiography indicated that global cardiac structure and function were comparable between SOCS3-CKO mice and WT mice.

### Sustained SOCS3 protein expression during myocardial IRI

**Fig 4:**
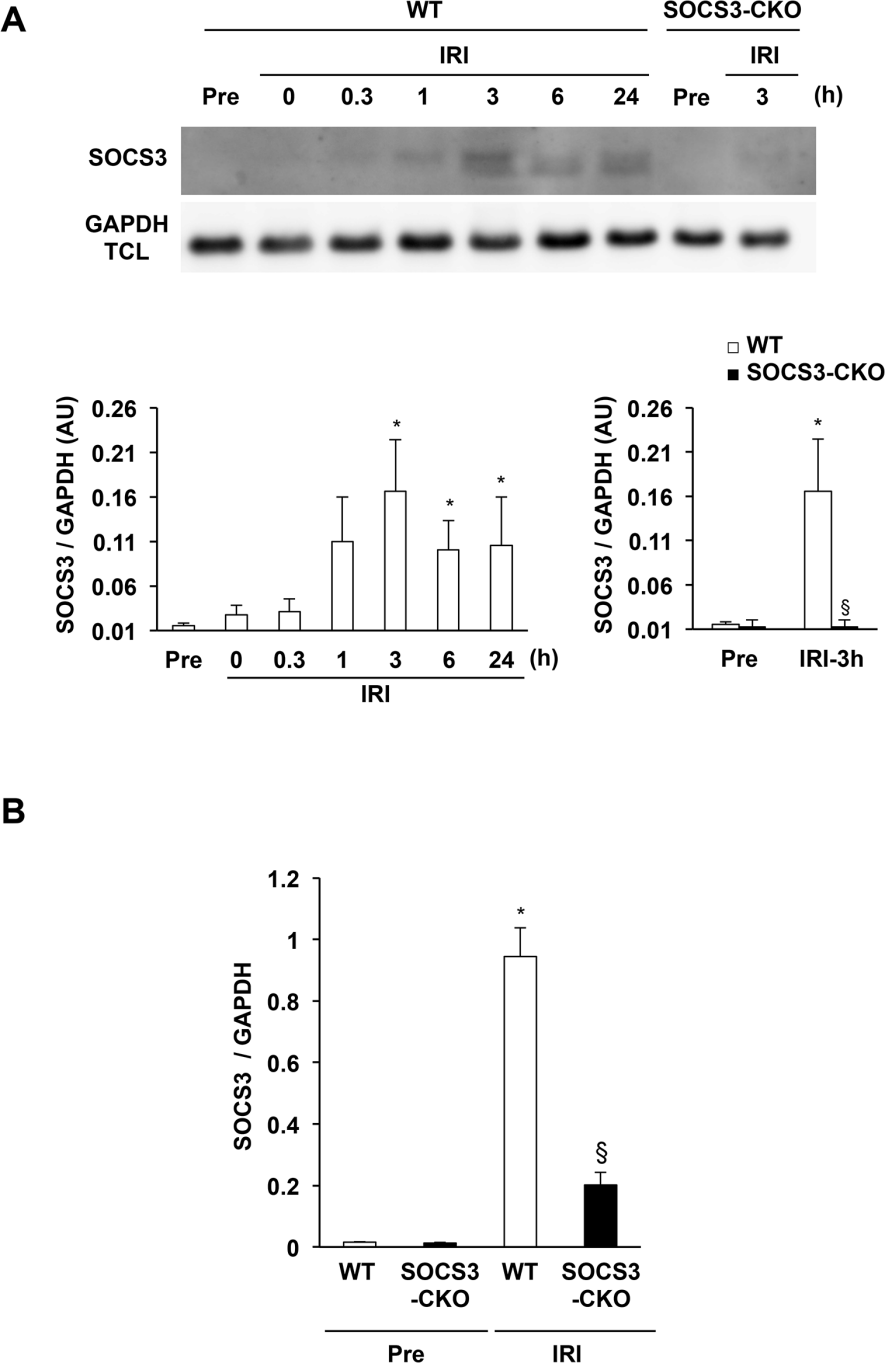
Early and sustained expression of SOCS3 protein during myocardial IRI. (**A**) Total cell lysates (TCL) were prepared from the left ventricle of wild-type (WT) and SOCS3-CKO mice at the indicated times after reperfusion and were immunoprecipitated with anti-SOCS3 antibodies. The immunoprecipitates and total cell lysates were subjected to western blotting and probed with antibodies against SOCS3 and GAPDH, respectively. The graphs represent the time course expression of SOCS3 protein in WT mice after reperfusion (left) and the quantitative difference between WT and SOCS3-CKO mice 3 h after reperfusion (right; n = 5 per group). *P < 0.05 vs. WT pre-ischemia. §*P* < 0.05 vs. WT 3 h after reperfusion. AU indicates arbitrary units; GAPDH, glyceraldehyde 3-phosphate dehydrogenase; IRI, ischemia reperfusion injury. (**B**) Real-time PCR analysis for SOCS3 mRNA expression in mouse hearts pre-ischemia or 3 h after reperfusion (n = 5 for each group). *P < 0.05 vs. pre-ischemia.

SOCS3 protein is difficult to detect due to its degradation through the ubiquitin–proteasome system [40]. To facilitate the detection of SOCS3 protein, we conducted immunoprecipitation and western blot analysis. The expression of SOCS3 protein was undetectable pre-ischemia, increased at 1 h and peaked 3 h after reperfusion, and was sustained for 24 h in WT mice (**Fig 4A**). In the SOCS3-CKO mice, SOCS3 protein expression was not detected 3 h after reperfusion (**Fig 4A**). Real-time PCR also revealed that, at 3 h after IRI, the expression of SOCS3 mRNA was increased in WT mice and was markedly reduced in SOCS3-CKO mice (**Fig 4B**).

### Reduced infarct size and preserved LV contraction in SOCS3-CKO mice after IRI

**Fig 5:**
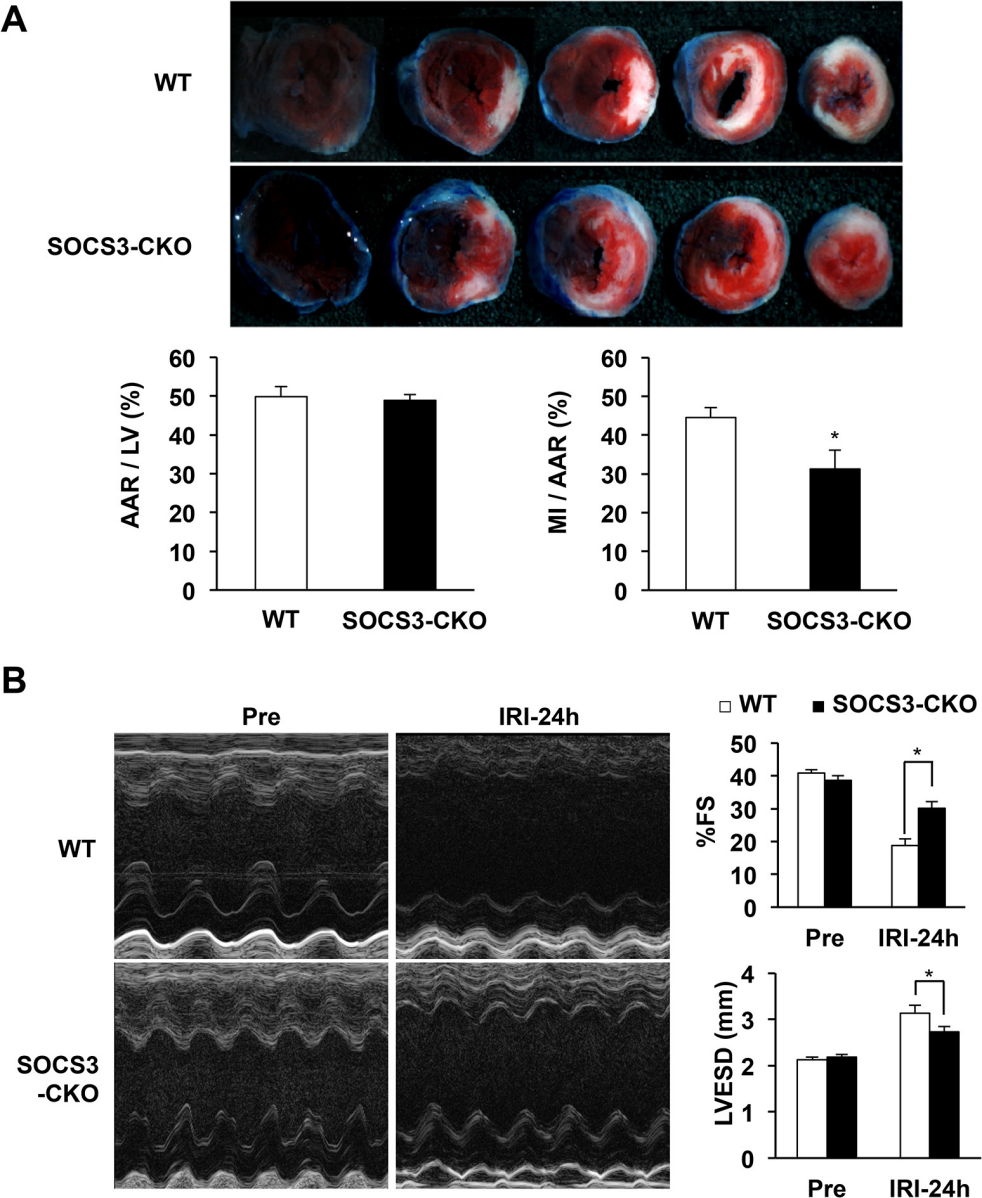
Reduced infarct size and preserved left ventricular systolic function after IRI in SOCS3-CKO mice. (**A**) Representative images of Evans blue and triphenyltetrazolium chloride (TTC) staining in wild-type (WT) and SOCS3-CKO mice 24 h after reperfusion (top) (n = 8 per group). The infarct size of the left ventricle (LV) was expressed as a percentage of the area at risk (AAR) of each group (left). The graphs show quantification of AAR/LV and infarct area/AAR. *P < 0.05 vs. WT mice. (**B**) Echocardiography was performed pre-ischemia and 24 h after reperfusion (n = 8 per group). Pooled data for echocardiographic measurements in WT and SOCS3-CKO mice. *P < 0.05 vs. WT mice. FS indicates fractional shortening; LVESD, left ventricular end systolic dimension; IRI, ischemia reperfusion injury.

**Fig 6:**
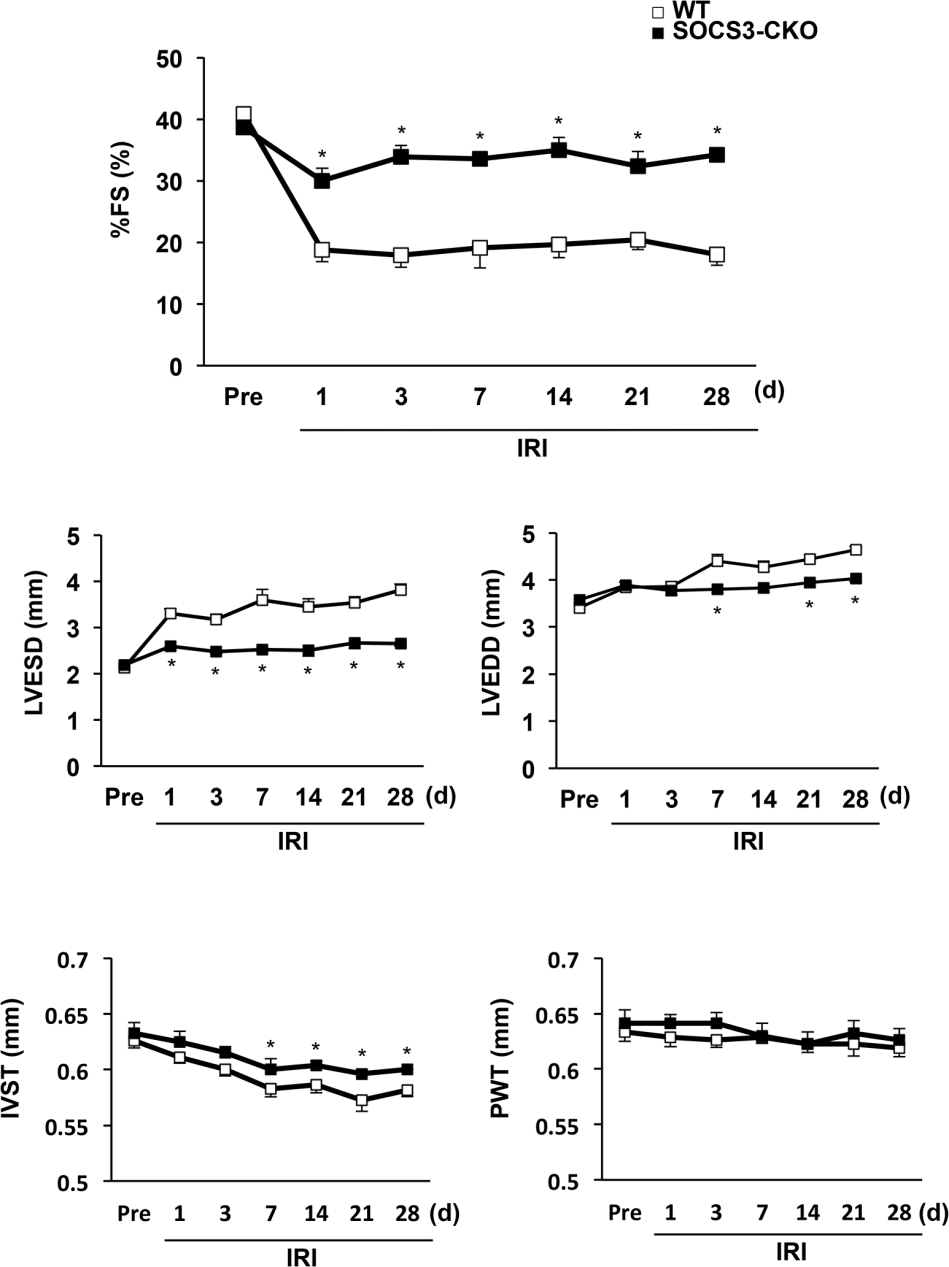
Echocardiography measurements in wild-type (WT) and cardiac-specific SOCS3-CKO mice during the chronic phase. Echocardiography was performed at pre-ischemia and from day 1 to day 28 after reperfusion (n = 8 per group). Pooled data for echocardiographic measurements in WT and SOCS3-CKO mice. *P < 0.05 vs. WT mice. FS indicates fractional shortening; LVESD, left ventricular end systolic dimension; LVEDD, left ventricular end diastolic dimension; IVST, interventricular septum thickness; PWT, posterior left ventricular wall thickness.

We employed Evans blue and TTC double staining 24 h after reperfusion to determine the infarct area; AAR, which indicates the ischemic area; and the normally perfused area of LV. Infarct size and ischemic size were defined, respectively, as the weight ratios of infarct area to AAR and AAR to LV. Infarct size was significantly reduced in the hearts of SOCS3-CKO compared with WT mice, even though the ischemic size of the two groups were comparable (**Fig 5A**). Echocardiographic assessment revealed that the percent fractional shortening was greater and the LV end-systolic dimension was smaller in SOCS3-CKO than in WT mice (**Fig 5B**). The LV end-diastolic dimension, interventricular septum thickness, and posterior LV wall thickness were comparable between SOCS3-CKO and WT mice 24 h after reperfusion (**S1 Fig**). During the chronic phase after reperfusion, the LV end-systolic dimension was gradually (not significantly) increased and the LV end-diastolic dimension was significantly increased in WT mice (**Fig 6**). In contrast, percent fractional shortening, LV end-systolic dimension, and LV end-diastolic dimension were preserved in SOCS3-CKO mice (**Fig 6**). The interventricular septum was significantly thicker in SOCS3-CKO mice than in WT mice from day 7 to 28 after reperfusion (**Fig 6**).

### Sustained activation of cardioprotective signaling molecules in SOCS3-CKO mice during IRI

**Fig 7:**
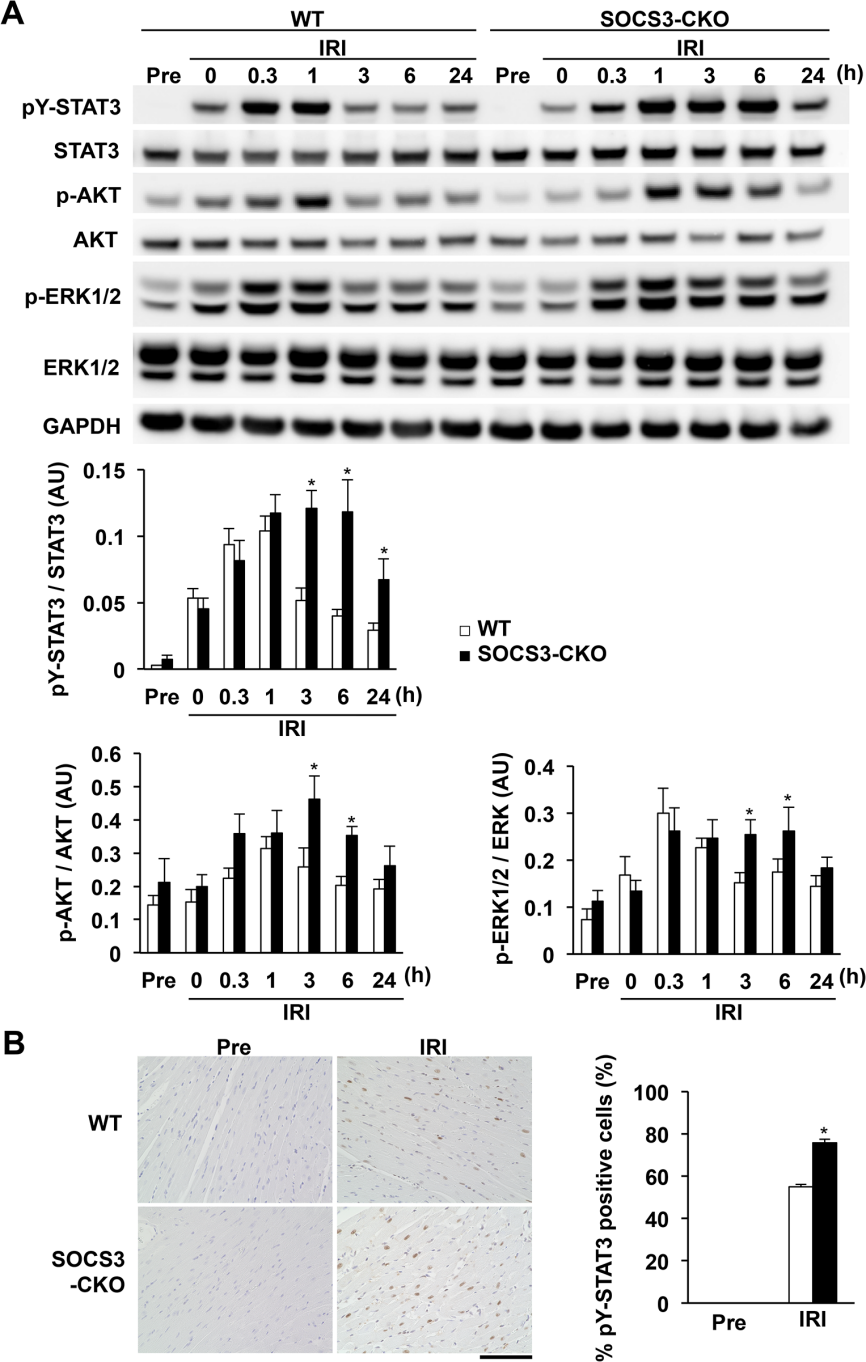
(A) Sustained activation of cardioprotective signaling molecules in SOCS3-CKO mice during IRI. Western blot analysis of total cell lysates prepared from the left ventricle of WT or SOCS3-CKO mice at the indicated times after reperfusion. The blots were probed using antibodies against tyrosine-phosphorylated STAT3 (pY-STAT3), STAT3, phosphorylated AKT (p-AKT), AKT, phosphorylated ERK1/2 (p-ERK1/2), and GAPDH. The graphs represent quantitative differences in the ratios of the expression of pY-STAT3 to STAT3, p-AKT to AKT, and p-ERK1/2 to ERK1/2 (n = 6 per group). *P < 0.05 vs. WT mice. AU, arbitrary units; IRI, ischemia reperfusion injury. (**B**) Immunostaining of tyrosine-phosphorylated STAT3 (pY-STAT3) in hearts pre-ischemia or 3 h after reperfusion (n = 5 for each group). Representative photographs of the hearts from each group are shown. Values are expressed as the percentage of pY-STAT3–positive cells in the hearts pre-ischemia or 6 h after reperfusion. *P < 0.05 vs. pre-ischemia. Scale bar = 100 μm. AU = arbitrary units.

Next, we performed western blot analysis to compare the activation of cardioprotective signaling pathways, including STAT3, AKT, and ERK1/2 during IRI in WT and SOCS3-CKO mice. STAT3 phosphorylation levels were similar between SOCS3-CKO and WT mice 1 h after reperfusion (**Fig 7A**). STAT3 phosphorylation in WT mice decreased from 3 h to 24 h after reperfusion (**Fig 7A**). In contrast, STAT3 phosphorylation levels peaked in SOCS3-CKO mice by 1 h and were sustained until 6 h after reperfusion, and STAT3 phosphorylation was significantly higher than in WT mice 24 h after reperfusion (**Fig 7A**). Immunohistochemical staining revealed that, at 6 h post-IRI, the number of pY-STAT3–positive cells was significantly greater in hearts from SOCS3-CKO mice than in those from WT mice (**Fig 7B**). AKT and ERK1/2 phosphorylation levels in SOCS3-CKO mice increased similarly 1 h after reperfusion compared with their levels in WT mice, and they were significantly higher than those of WT mice up to 6 h after reperfusion (**Fig 7A**). Thus, the IRI-induced activation of cardioprotective signaling molecules was sustained for a longer duration in SOCS3-CKO mice than in WT mice.

### Reduction of myocardial apoptosis in SOCS3-CKO mice after IRI

**Fig 8:**
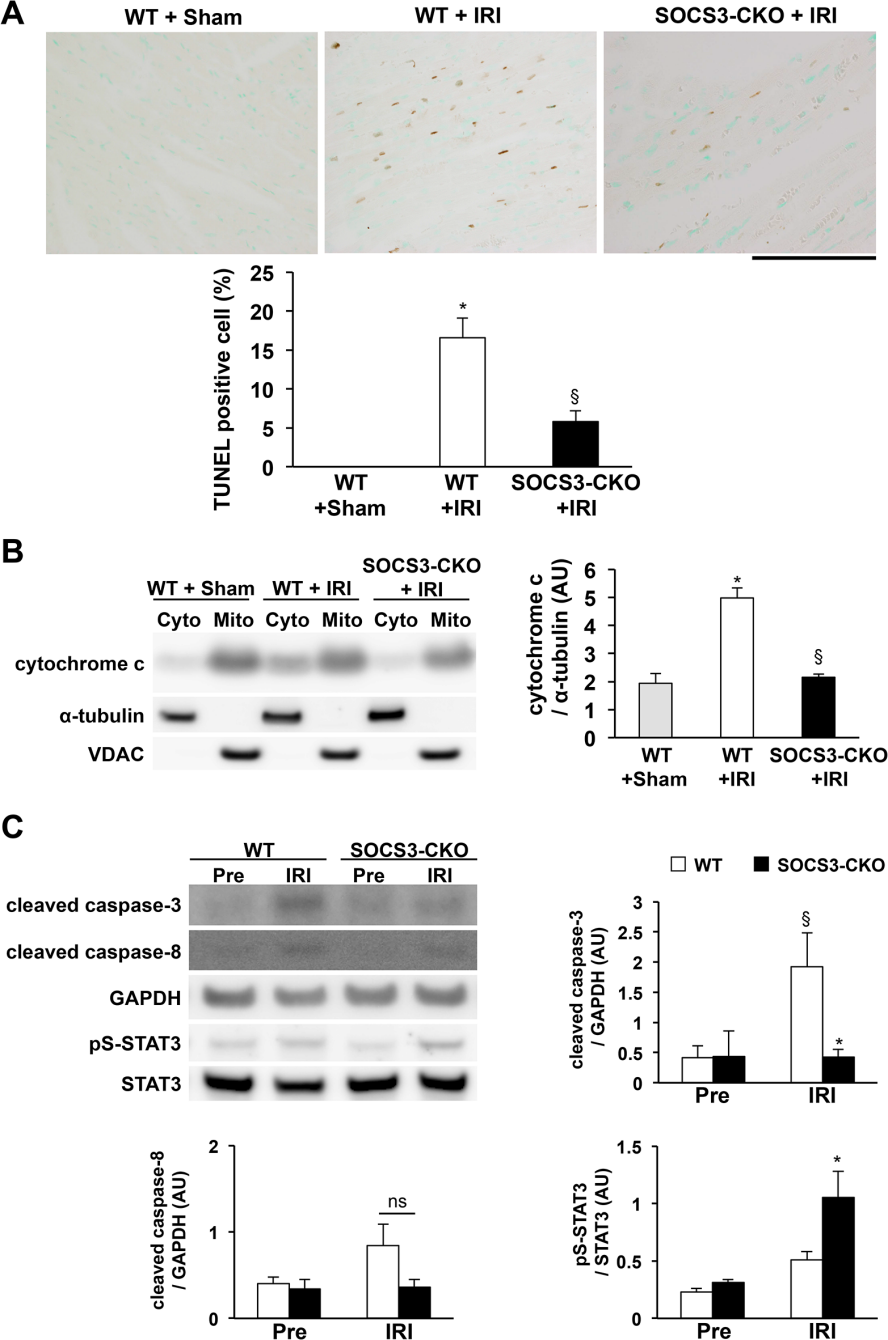
Reduction of apoptosis and mitochondrial cytochrome c release in SOCS3-CKO mice during IRI. (**A**) Terminal deoxynucleotidyl transferase dUTP nick end labeling (TUNEL) assays of hearts from wild-type (WT) or SOCS3-CKO mice 6 h after reperfusion (n = 5 per group). Representative images of the ischemic areas of hearts from each group. The graph shows the percentage of TUNEL-positive cells. *P < 0.05 vs. WT sham, §*P* < 0.05 vs. WT IRI. (**B**) The cytosolic (Cyto) and mitochondrial (Mito) fractions were prepared from the left ventricles of WT or SOCS3-CKO mice 6 h after reperfusion and subjected to western blotting using an antibody against cytochrome c. The cytosolic marker α-tubulin and the mitochondrial marker VDAC served as internal controls for WT mice (n = 6 per group). The graph represents quantitative differences in cytochrome c expression. *P < 0.05 vs. WT sham, §*P* < 0.05 vs. WT IRI. AU, arbitrary units; IRI, ischemia reperfusion injury. (**C**) Total cell lysates were prepared from the left ventricles of wild-type (WT) or SOCS3-CKO mice pre-ischemia or 6 h after reperfusion, and western blots were probed with antibodies raised against cleaved caspase 8, cleaved caspase 3, and serine-phosphorylated STAT3 (pS-STAT3). The graphs represent quantitative differences in expression among cleaved caspase 8, cleaved caspase 3, and PS-STAT3 (n = 5 per group). §*P* < 0.05 vs. WT pre-ischemia, *P < 0.05 vs. WT 6 h after IRI. AU, arbitrary units; IRI, ischemia reperfusion injury.

**Fig 9:**
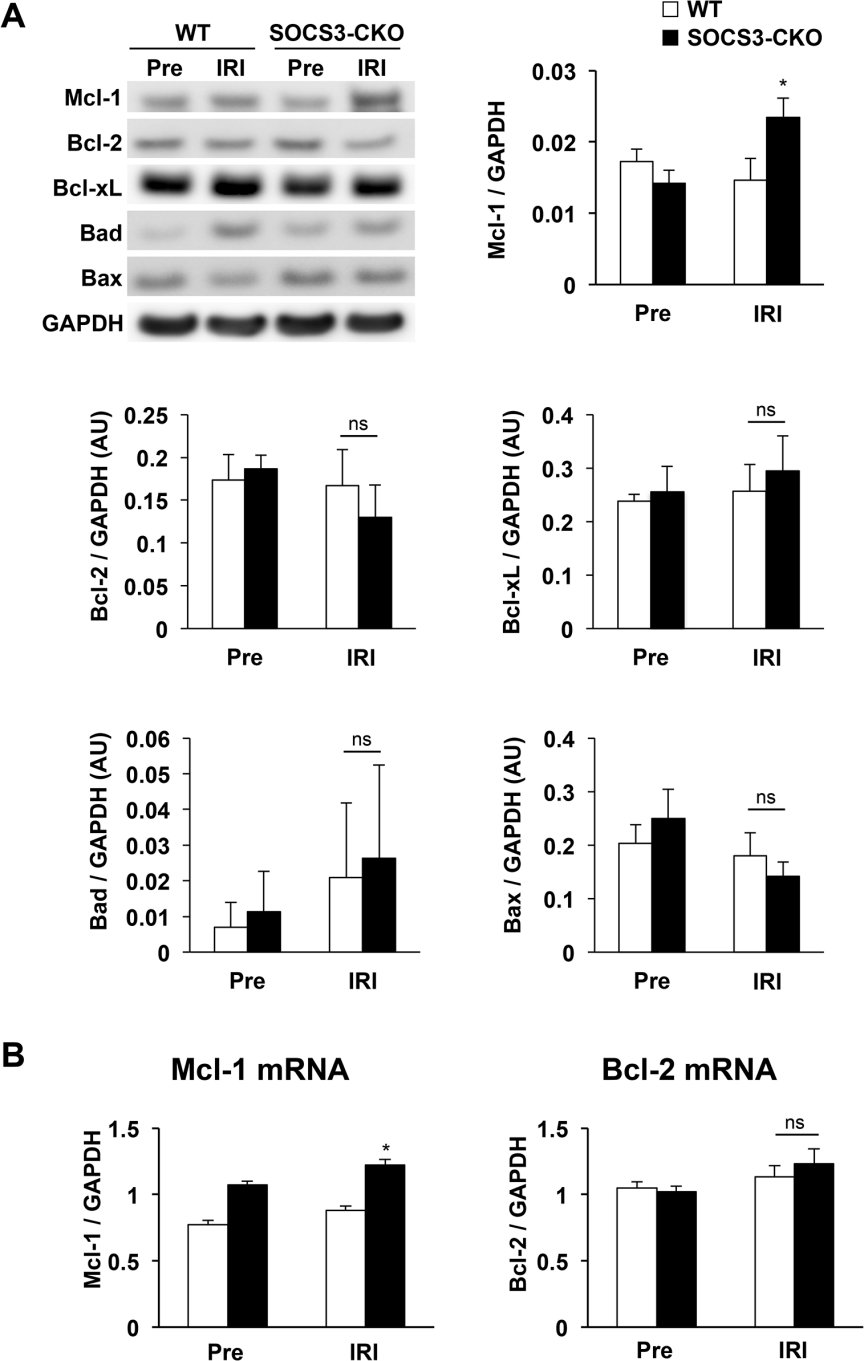
(A) Increased expression of anti-apoptotic Mcl-1 after IRI in SOCS3-CKO mice. Total cell lysates were prepared from the left ventricles of wild-type (WT) or SOCS3-CKO mice pre-ischemia or 6 h after reperfusion, and western blots were probed with antibodies raised against Mcl-1, Bcl-xL, Bcl-2, Bad, and Bax. The graphs represent quantitative differences in expression among Mcl-1, Bcl-xL, Bcl-2, Bad, and Bax (n = 5 per group). *P < 0.05 vs. WT IRI. AU, arbitrary units; IRI, ischemia reperfusion injury. (**B**) Real-time PCR analysis of Mcl1 and Bcl2 mRNA expression in the hearts pre-ischemia and 3 h after reperfusion (n = 5 for each group). *P < 0.05 vs. WT after reperfusion.

The apoptosis of cardiomyocytes is centrally involved in the development of myocardial injury after IRI [6–8]. We performed TUNEL assay to measure the number of apoptotic cells 6 h after reperfusion. The number of TUNEL-positive cells was markedly reduced in SOCS3-CKO compared with WT mice (**Fig 8A**). Cytochrome c release from the mitochondria into the cytosol was decreased after IRI in SOCS3-CKO mice compared with WT mice (**Fig 8B**). Western blot analysis revealed that, after IRI, expression of cleaved caspase 8 was comparable between WT and SOCS3-CKO mice, while the expression of cleaved caspase 3 was lower in hearts from SOCS3-CKO mice than in those from WT mice (**Fig 8C**). Because activation of STAT3 at serine 727 has been indicated to regulate a metabolic function in mitochondria and act in the regulation of survival and apoptosis [41], we used western blotting to measure pS-STAT3. We found that, after IRI, the expression of pS-STAT3 was greater in hearts from SOCS3-CKO mice than in hearts from WT mice (**Fig 8C**). We determined the expression levels of mitochondria-associated pro- and anti-apoptotic molecules in the hearts of SOCS3-CKO and WT mice 6 h after reperfusion. Although the expression of anti-apoptotic Bcl-2 and Bcl-xL and pro-apoptotic Bad and Bax expression were comparable between SOCS3-CKO and WT mice, Mcl-1 (an anti-apoptotic member of the Bcl2 family) was expressed at a significantly higher level in SOCS3-CKO mice than in WT mice 6 h after reperfusion (**Fig 9A**). Real-time PCR also revealed that expression of Mcl1 mRNA, but not Bcl2 mRNA, was greater in SOCS3-CKO mice than in WT mice after reperfusion (**Fig 9B**).

### Augmentation of antioxidant gene expression and reduction of pro-inflammatory gene expression

**Fig 10:**
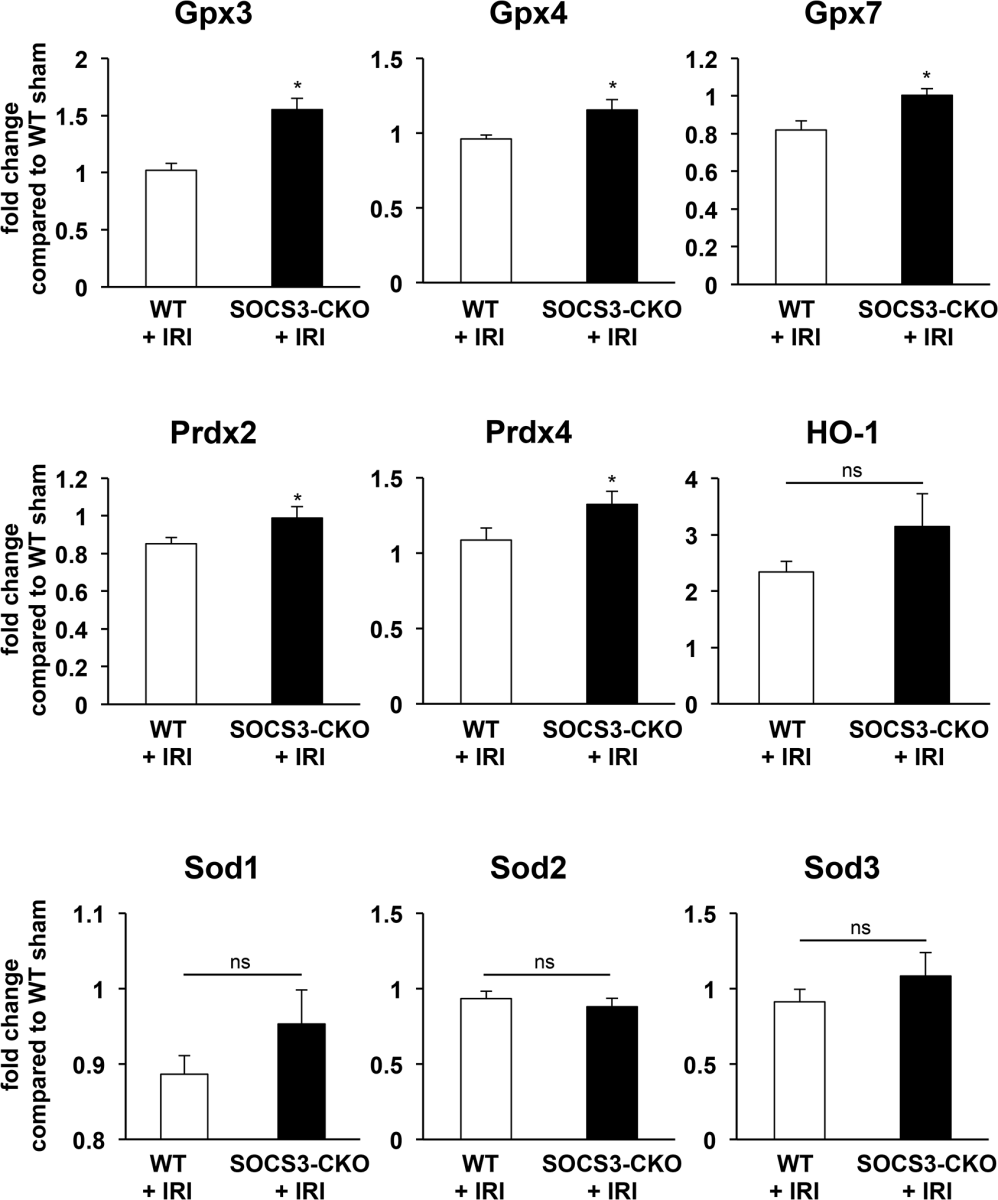
Expression of genes related to oxidative stress during myocardial IRI. mRNA was prepared from the left ventricles of WT or SOCS3-CKO mice after reperfusion and subjected to real-time PCR analysis regarding the indicated genes. Values normalized against glyceraldehyde 3-phosphate dehydrogenase (GAPDH) are expressed as fold change from the values of sham mice (n = 5 for each group). *P<0.05 versus WT sham. Gpx = glutathione peroxidase; Prdx = peroxiredoxin; HO = heme oxygenase; Sod = superoxide dismutase.

**Fig 11:**
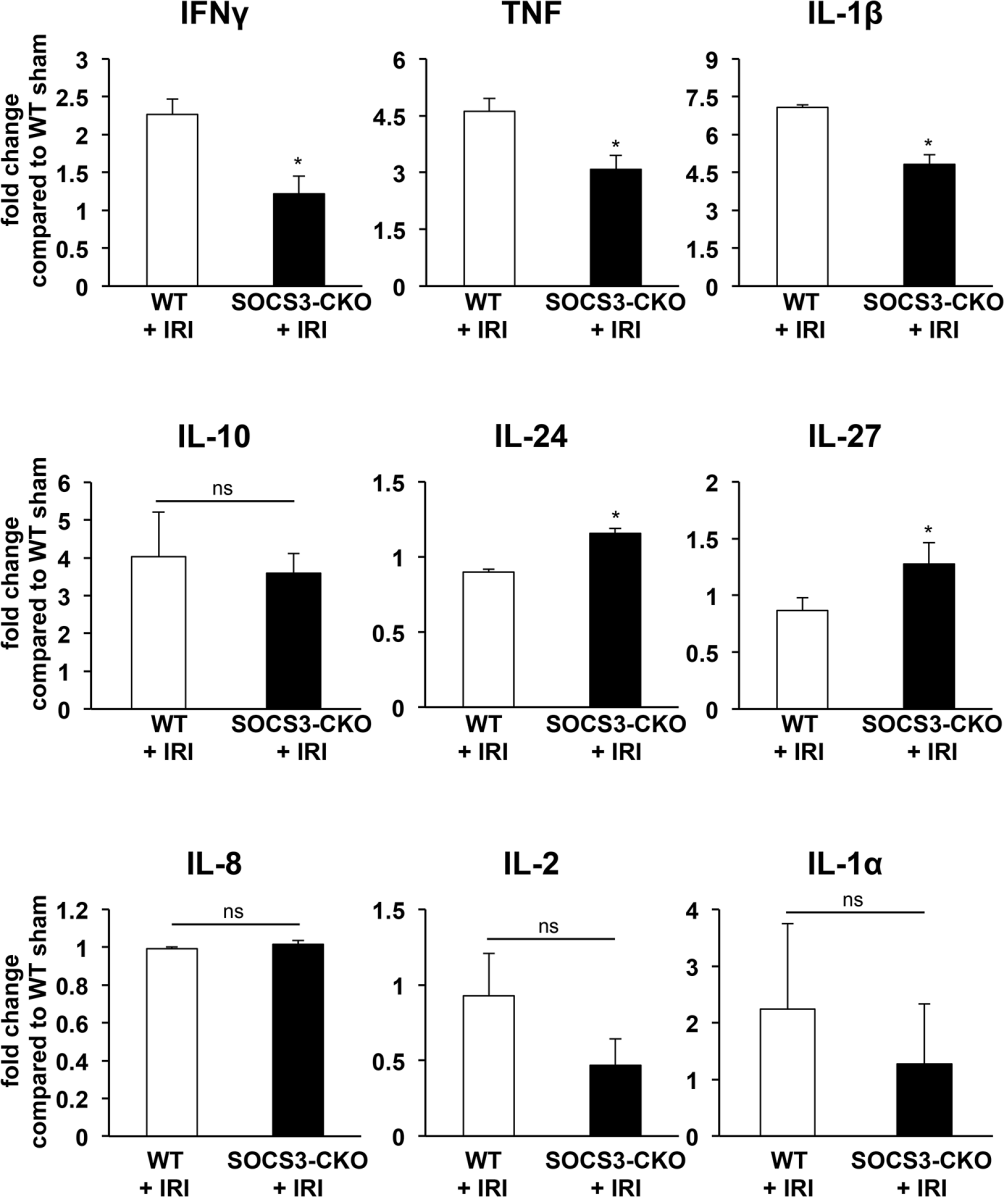
Expression of pro-inflammatory and anti-inflammatory cytokine genes during myocardial IRI. mRNA was prepared from the left ventricles of WT or SOCS3-CKO mice after reperfusion and subjected to real-time PCR analysis regarding the indicated genes. Values normalized against glyceraldehyde 3-phosphate dehydrogenase (GAPDH) are expressed as fold change from the values of sham mice (n = 5 for each group). *P<0.05 versus WT sham. IL = interleukin; IFN = interferon; TNF = tumor necrosis factor.

Because STAT3 is involved in the regulation of oxidative stress and inflammation that may affect apoptosis during myocardial IRI, we examined PCR array analysis for indications of oxidative stress and inflammation during myocardial IRI. The expression of some antioxidant enzymes, including glutathione peroxidase (Gpx)-3, Gpx4, Gpx7, peroxiredoxin (Prdx)-2, and Prdx4 was significantly lower in SOCS3-CKO mice than in WT mice after reperfusion (**Fig 10**). The expression of pro-inflammatory cytokines, including interferon-γ, tumor necrosis factor-α (TNFα), and IL-1β were significantly lower in SOCS3-CKO than in WT mice, and the expression of anti-inflammatory cytokines (including IL-24 and IL-27, but not IL-10) were greater in SOCS3-CKO than in WT mice after reperfusion (**Fig 11**).

## Discussion

In the present study, we attempted to determine the role of myocardial SOCS3, an intrinsic negative feedback regulator of the JAK-STAT pathway-activating cytokines, in the inhibitory mechanism of cardioprotective signaling molecules during myocardial IRI in mice. For this purpose, we determined the activation kinetics of cardioprotective signaling molecules STAT3, AKT, and ERK1/2. The activation of STAT3 peaked rapidly but was promptly suppressed after IRI, which correlated with the induction of SOCS3 expression for as long as 24 h after IRI in WT mice. Cardiac-specific deletion of SOCS3 induced the sustained activation of cardioprotective signaling molecules and inhibited myocardial apoptosis through the increased expression of anti-apoptotic Mcl-1, resulting in the prevention of myocardial IRI. These findings suggest that myocardial SOCS3 suppresses the activation of cardioprotective signaling molecules during IRI and that SOCS3 may be centrally involved in the development of myocardial IRI.

### Mechanism of inhibition of cardioprotective signaling molecules during myocardial IRI

Many lines of evidence suggest that STAT3 is one of the major cardioprotective signaling molecules against myocardial IRI [9–16]. However, previous reports do not focus on the inhibitory mechanism against the activation of STAT3 during myocardial IRI. McCormick et al. showed the transient activation of STAT3 within 2 h after reperfusion in an *in vivo* rat model of myocardial IRI; they suggested that a negative feedback mechanism inhibits the full activation of STAT3 [27]. In the present study, we precisely examined the activation kinetics of the cardioprotective signaling molecules STAT3, AKT, and ERK1/2 for up to 24 h after reperfusion. The levels of STAT3, AKT, and ERK1/2 phosphorylation were comparable between WT and SOCS3-CKO mice within 1 h after reperfusion (**Fig 7**), suggesting that the inhibitory mechanism was not active during this period. Thus, SOCS3 protein was not significantly induced until 1 h after reperfusion (**Fig 4**). From 3 to 24 h after reperfusion, the time course of SOCS3 protein expression paralleled the sustained reduction of STAT3 phosphorylation. Because the SOCS3 promoter contains a functionally important STAT3-binding elements [42], the induction of SOCS3 protein expression was dependent on the activation of STAT3 after reperfusion. Thus, SOCS3 is a STAT3-induced negative feedback regulator of the activation of STAT3 during myocardial IRI.

In contrast to the enhanced and prolonged phosphorylation of STAT3, the enhanced and prolonged phosphorylation of AKT and ERK1/2 is mild during myocardial IRI in SOCS3-CKO mice (**Figs 2 and 7**). It is well known that STAT3 is a specific downstream signaling molecule of JAK and is mainly activated by JAK-STAT–activating cytokines [28, 29]. Meanwhile, AKT and ERK1/2 are activated not only by JAK-STAT–activating cytokines, but also by a variety of cytokines and growth factors that do not activate JAK [43, 44]. We previously reported that the forced expression of SOCS3 completely inhibits the LIF-induced activation of AKT, ERK1/2, and STAT3 in the cardiomyocytes [33]. Because SOCS3 is a JAK binding protein and strongly suppresses JAK kinase activity [28, 29], SOCS3 is able to inhibit the activation of all JAK downstream signaling molecules, including AKT and ERK1/2, but is not able to inhibit the activation of AKT and ERK1/2 by cytokines and growth factors that do not utilize JAK. A wide range of cytokines and growth factors are induced during myocardial IRI [4, 5]. SOCS3 is able to inhibit the activation of AKT and ERK1/2 by JAK-STAT–activating cytokines, but is not able to inhibit the activation of AKT and ERK1/2 by cytokines and growth factors that do not utilize JAK during IRI. This is the reason for the mild enhancement and prolongation of AKT and ERK1/2 activation during myocardial IRI in SOCS3-CKO mice. Further, Ruan et al. determined the role of myocardial phosphatase and tensin homolog on chromosome ten (PTEN), an important negative regulator of the PI3 kinase-AKT pathway, in myocardial IRI [45]. They demonstrated greater activation of AKT and ERK1/2 and reduced myocardial IRI in cardiac-specific PTEN knockout mice [45]. Thus, other inhibitors such as PTEN may contribute to the inhibitory mechanism of AKT and ERK1/2 activation during myocardial IRI.

### Inhibition of IRI-induced myocardial apoptosis by cardiac-specific deletion of SOCS3

Apoptosis plays a major role in the development of myocardial IRI [46]. For example, animal studies have shown that myocardial STAT3 is a potent anti-apoptotic, pro-survival signaling molecule during IRI [9–16]. Consistent with previous reports, we show here that the cardiac-specific deletion of SOCS3 induced a sustained activation of STAT3 and reduced myocardial apoptosis that prevented IRI in mice. However, the expression levels of anti-apoptotic Bcl2 and Bcl-xL and pro-apoptotic Bad and Bax were comparable between WT and SOCS3-CKO mice, even though STAT3 regulates their expression [16]. Because SOCS3 inhibits multiple cytokines and growth factors that utilize the JAK kinase to transduce their signals [28–30], the deletion of SOCS3 may enhance the action of multiple cytokines and growth factors. Furthermore, the deletion of SOCS3 may enhance multiple JAK downstream signaling pathways including STAT3, AKT, and ERK1/2. This may explain the comparable expression levels of molecules involved in apoptosis between WT and SOCS3-CKO mice. Among molecules involved in apoptosis, we found that the expression of the anti-apoptotic Bcl2 family member Mcl-1 was significantly upregulated in SOCS3-CKO mice compared with WT mice. Bolli et al. reported that ischemic preconditioning increased the expression of Mcl-1 in WT mouse hearts, which was absent in cardiac-specific STAT3 knockout mice, suggesting that Mcl-1 is a STAT3 target gene [47]. The loss of Mcl-1 expression promotes the induction of apoptosis in a variety of other tissues [48]. Recently, Wang et al. reported that cardiac-specific Mcl-1 knockout mice exhibit myocardial cell apoptosis and mitochondrial abnormalities leading to dilated cardiomyopathy [49], which indicates a critical role for Mcl-1 in the prevention of myocardial cell apoptosis. Taken together with our present findings, we suggest that the upregulated expression of Mcl-1 during IRI may play an important role in preventing myocardial apoptosis in SOCS3-CKO mice.

It is well known that oxidative stress and inflammation are centrally involved in the myocardial damage caused by myocardial IRI. For example, Dabkowski et al. reported that mitochondria-specific transgenic overexpression of the antioxidant enzyme Gpx4 attenuates myocardial damage during IRI [50]. Suzuki et al. reported that the overexpression of IL-1 receptor antagonist attenuates myocardial apoptosis during IRI [51]. In the present study, some antioxidant enzymes (including Gpx4) were increased and main pro-inflammatory cytokines (including IL-1β were decreased in SOCS3-CKO mice after reperfusion. These increases of antioxidant enzymes and reductions of pro-inflammatory cytokines may contribute to the attenuation of myocardial apoptosis during IRI in SOCS3-CKO mice.

### SOCS3 is a therapeutic target for myocardial IRI

We previously reported that the expression of SOCS3 mRNA is rapidly induced (within 30 min) in cytokine-stimulated cardiomyocytes or pressure-overloaded mouse hearts, suggesting that SOCS3 is an early response gene [33]. In the present study, we detected the expression of SOCS3 protein 3 h after reperfusion, which unexpectedly persisted for 24 h. This SOCS3 protein expression paralleled the sustained reduction of STAT3 activation during IRI. Furthermore, we demonstrated previously that the adenoviral expression of SOCS3 completely inhibits the cytokine-induced activation of STAT3, AKT, and ERK1/2 in cardiomyocytes [33, 34]. Thus, SOCS3 is a potent inhibitor of the JAK-STAT signaling pathway, is persistently expressed during IRI, and suppresses IRI-induced STAT3 activation. Therefore, because cardiac-specific deletion of SOCS3 prevented myocardial apoptosis and dysfunction during IRI, myocardial SOCS3 may represent a key factor that exacerbates the development of IRI.

Resistance to cytokines limits their intrinsic efficacy. We showed previously that SOCS3 confers resistance to cytokines (e.g., interferon, leptin, and cardiotrophin-1) that utilize the JAK-STAT pathway to transduce their signals [52–54]. Despite the strong experimental evidence supporting a real cardioprotective effect of JAK-STAT–activating cytokines, including erythropoietin and G-CSF, in animal models of myocardial IRI, clinical trials have failed to translate a benefit into the clinical setting [55]. In the present study, we showed that the SOCS3 protein was rapidly and persistently expressed during myocardial IRI. In addition SOCS3 is induced not only by myocardial insults, but also by administered cytokine itself. Therefore, IRI-induced SOCS3 may reduce the effect of cytokine therapy in patients with acute myocardial infarction. Small-molecule antagonists of SOCS3 or tissue-specific vector delivery of SOCS3 inhibitor may represent clinically valuable strategies to enhance the protective effect of both endogenous and administered JAK-STAT–activating cytokines during myocardial IRI.

In the present study, we show that activation of the cardioprotective signaling molecules STAT3, AKT, and ERK1/2 rapidly peaked and was promptly suppressed during IRI. This suppression paralleled the induction of SOCS3 expression after the onset of IRI in WT mice. In SOCS3-CKO mice, STAT3, AKT, and ERK1/2 phosphorylation was sustained, myocardial apoptosis and injury were prevented, and the expression of the anti-apoptotic Bcl-2 family member Mcl-1 was augmented. Thus, the deletion of myocardial SOCS3 induced the sustained activation of cardioprotective signaling molecules including STAT3 and prevented myocardial apoptosis and injury during IRI. Our findings suggest that SOCS3 may represent a key factor that exacerbates the development of myocardial IRI.

## Supporting Information

S1 Fig Echocardiography of wild-type (WT) and cardiac-specific suppressor of cytokine signaling-3 knockout (SOCS3-CKO) mice. Echocardiography was performed pre-ischemia and 24 h after reperfusion (n = 8 per group). The LV end-diastolic dimension (LVEDD), interventricular septum thickness (IVST), and posterior left ventricular wall thickness (PWT) were comparable between the two groups at 24 h after reperfusion.
(TIF)

S2 Fig Heart sections from 10-week-old wild-type (WT) and SOCS3-CKO mice stained with hematoxylin/eosin and Mallory-Azan are shown. (JPG)

S3 Fig Full unedited blots for Fig 2. Blots were probed using antibodies against tyrosine-phosphorylated STAT3 (pY-STAT3), STAT3, phosphorylated AKT (p-AKT), AKT, phosphorylated ERK1/2 (p-ERK1/2), and GAPDH.
(JPG)

S4 Fig Full unedited blots for Fig 4. Blots were probed using antibodies against SOCS3 and GAPDH.
(JPG)

S5 Fig Full unedited blots for Fig 7. Blots were probed using antibodies against tyrosine-phosphorylated STAT3 (pY-STAT3), STAT3, phosphorylated AKT (p-AKT), AKT, phosphorylated ERK1/2 (p-ERK1/2), and GAPDH.
(JPG)

S6 Fig Full unedited blots for Fig 8. Blots were probed using antibodies against cytochrome c, α-tubulin, VDAC, GAPDH, cleaved caspase 8, cleaved caspase 3, serine-phosphorylated STAT3 (pS-STAT3), and STAT3.
(JPG)

S7 Fig Full unedited blots for Fig 9. Blots were probed using antibodies against myeloid cell leukemia-1 (Mcl-1), Bcl-xL, Bcl-2, Bad, Bax, and GAPDH.
(JPG)
