# Supplementary figures and images for: Cardiac-Specific SOCS3 Deletion Prevents In Vivo Myocardial Ischemia Reperfusion Injury through Sustained Activation of Cardioprotective Signaling Molecules
Source: PLoS One. 2015 May 26;10(5):e0127942. doi: 10.1371/journal.pone.0127942 (PMC4444323; doi:10.1371/journal.pone.0127942)

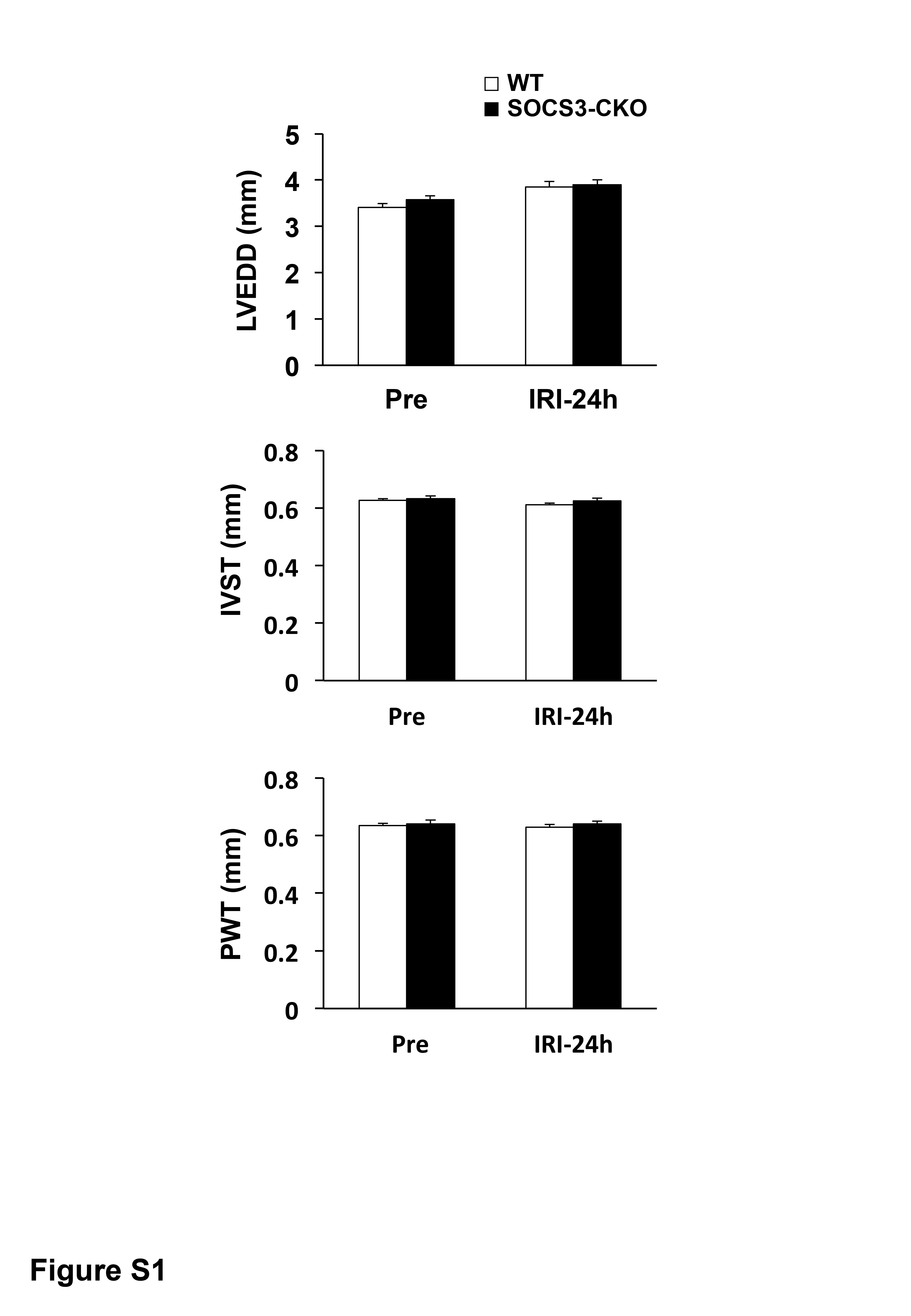

Supplement: S1 Fig — Echocardiography was performed pre-ischemia and 24 h after reperfusion (n = 8 per group). The LV end-diastolic dimension (LVEDD), interventricular septum thickness (IVST), and posterior left ventricular wall thickness (PWT) were comparable between the two groups at 24 h after reperfusion. (TIF) [file pone.0127942.s001.tif]

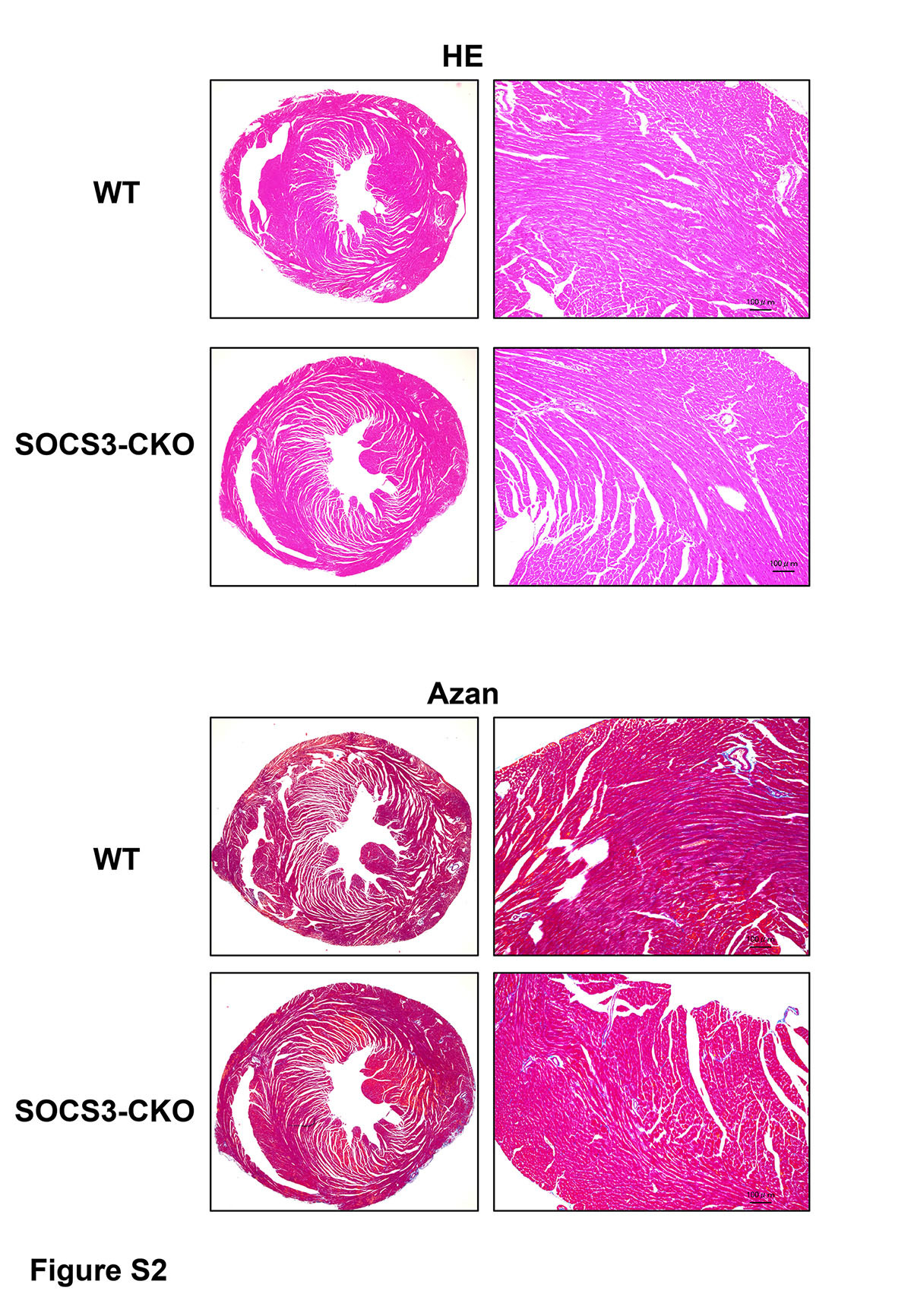

Supplement: S2 Fig — (JPG) [file pone.0127942.s002.jpg]

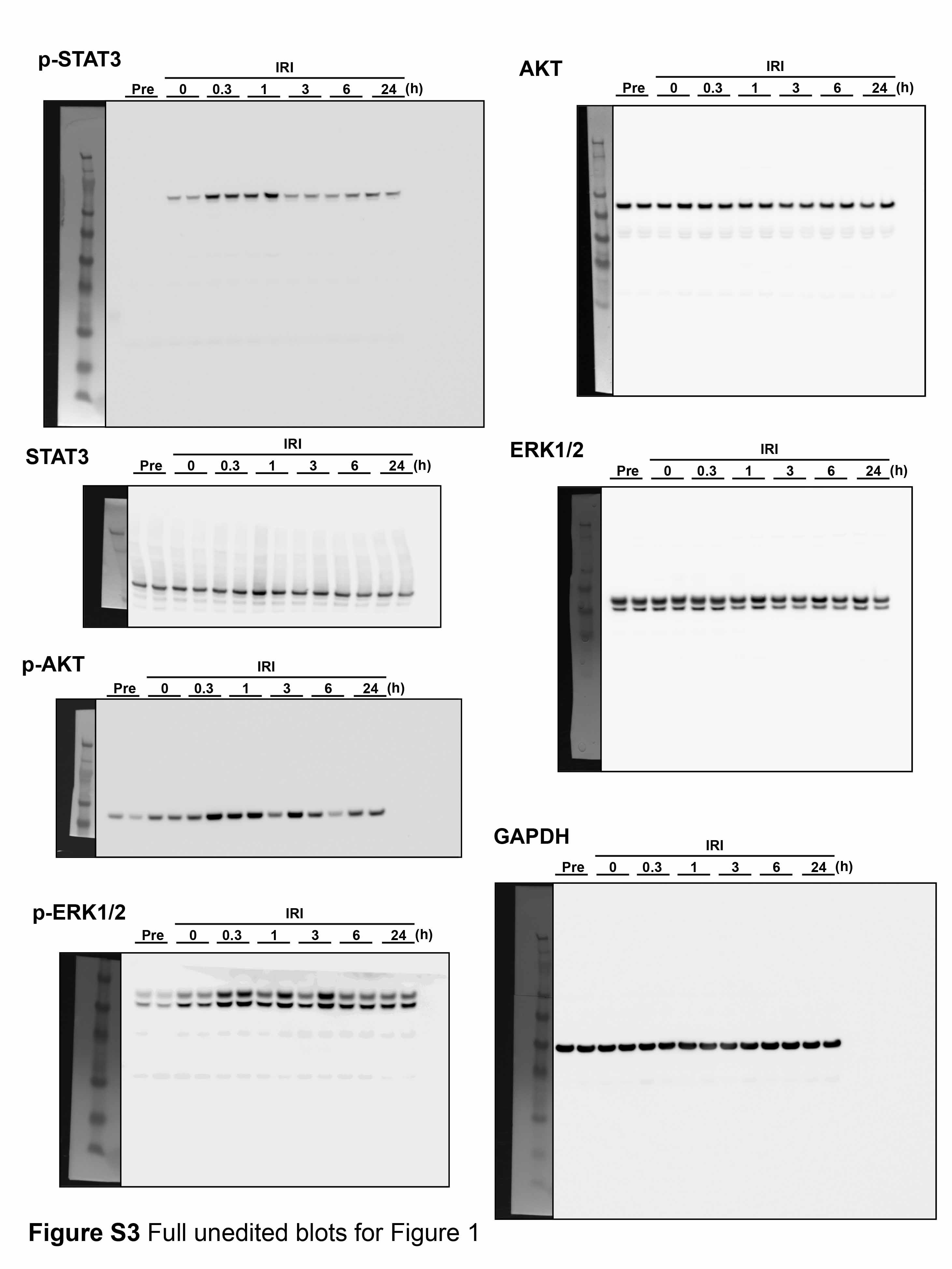

Supplement: S3 Fig — Blots were probed using antibodies against tyrosine-phosphorylated STAT3 (pY-STAT3), STAT3, phosphorylated AKT (p-AKT), AKT, phosphorylated ERK1/2 (p-ERK1/2), and GAPDH. (JPG) [file pone.0127942.s003.jpg]

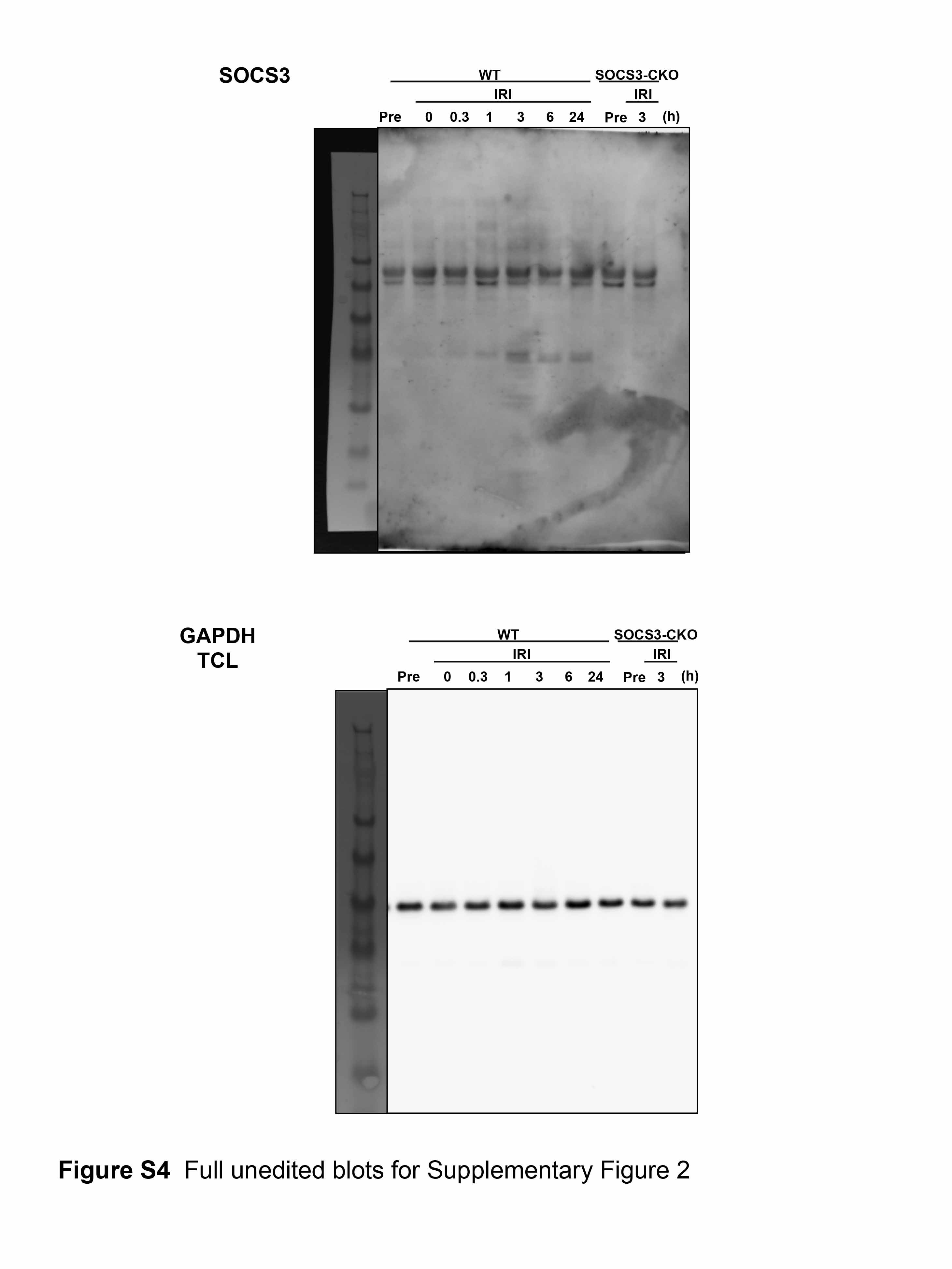

Supplement: S4 Fig — Blots were probed using antibodies against SOCS3 and GAPDH. (JPG) [file pone.0127942.s004.jpg]

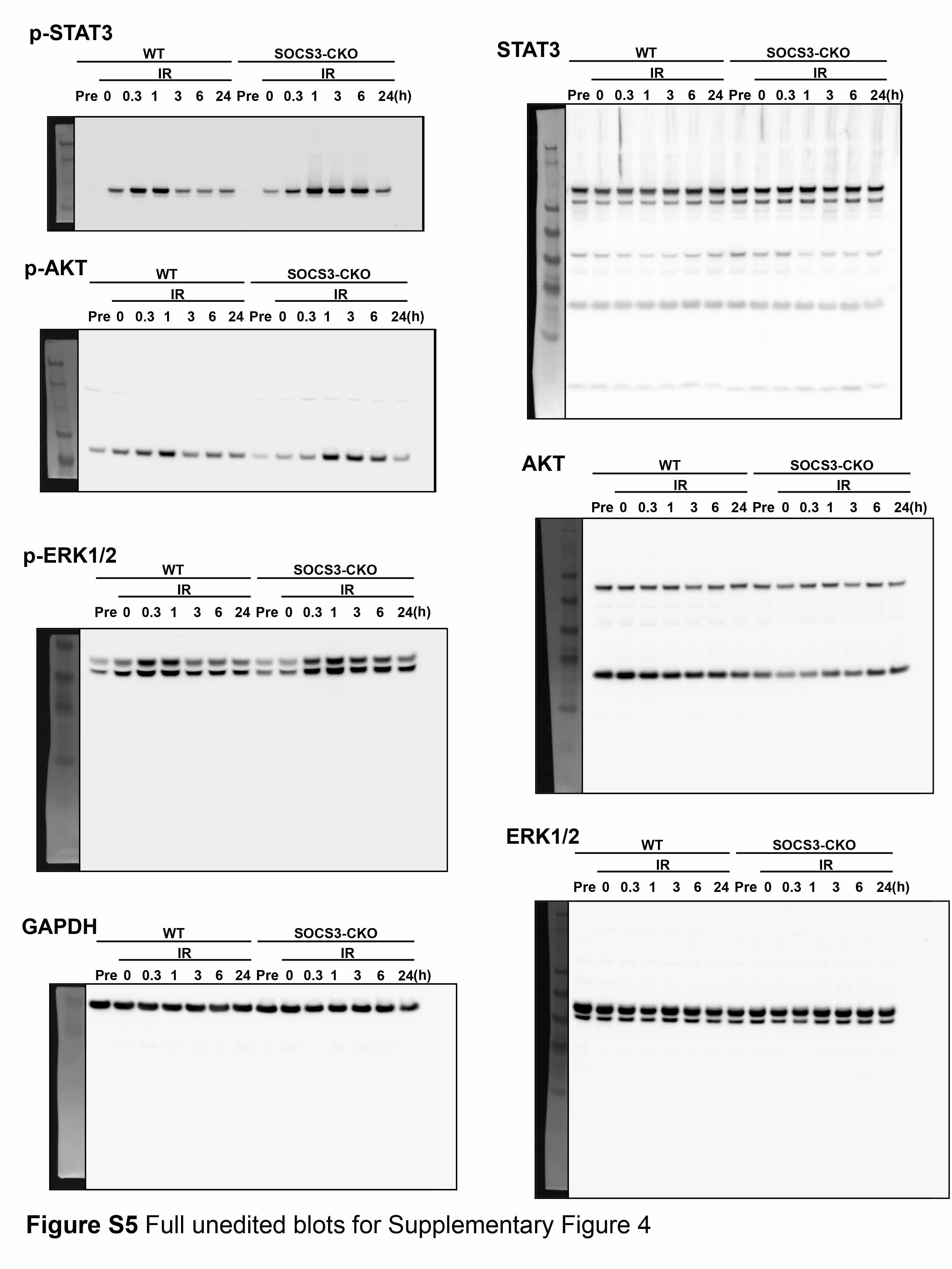

Supplement: S5 Fig — Blots were probed using antibodies against tyrosine-phosphorylated STAT3 (pY-STAT3), STAT3, phosphorylated AKT (p-AKT), AKT, phosphorylated ERK1/2 (p-ERK1/2), and GAPDH. (JPG) [file pone.0127942.s005.jpg]

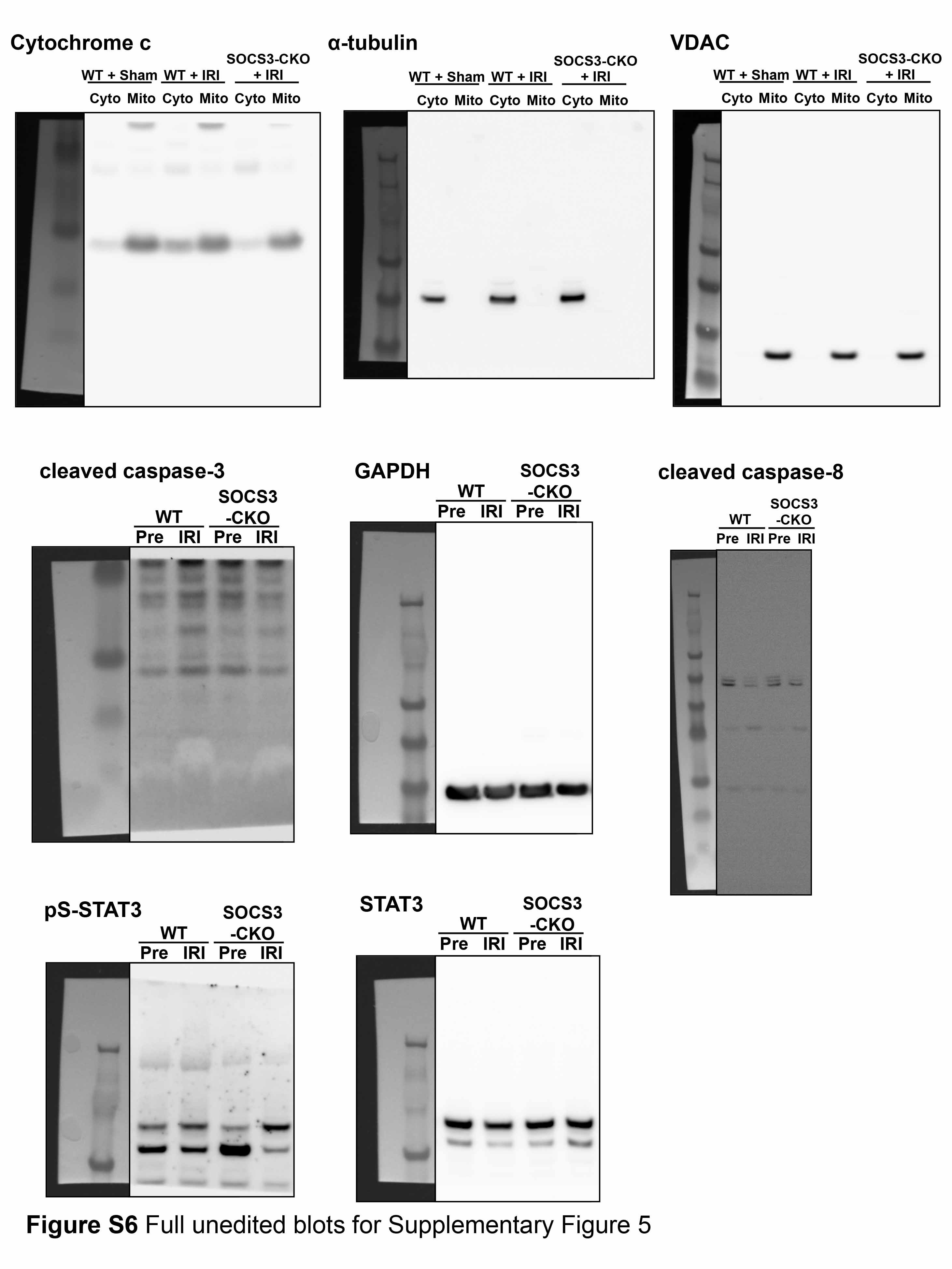

Supplement: S6 Fig — Blots were probed using antibodies against cytochrome c, α-tubulin, VDAC, GAPDH, cleaved caspase 8, cleaved caspase 3, serine-phosphorylated STAT3 (pS-STAT3), and STAT3. (JPG) [file pone.0127942.s006.jpg]

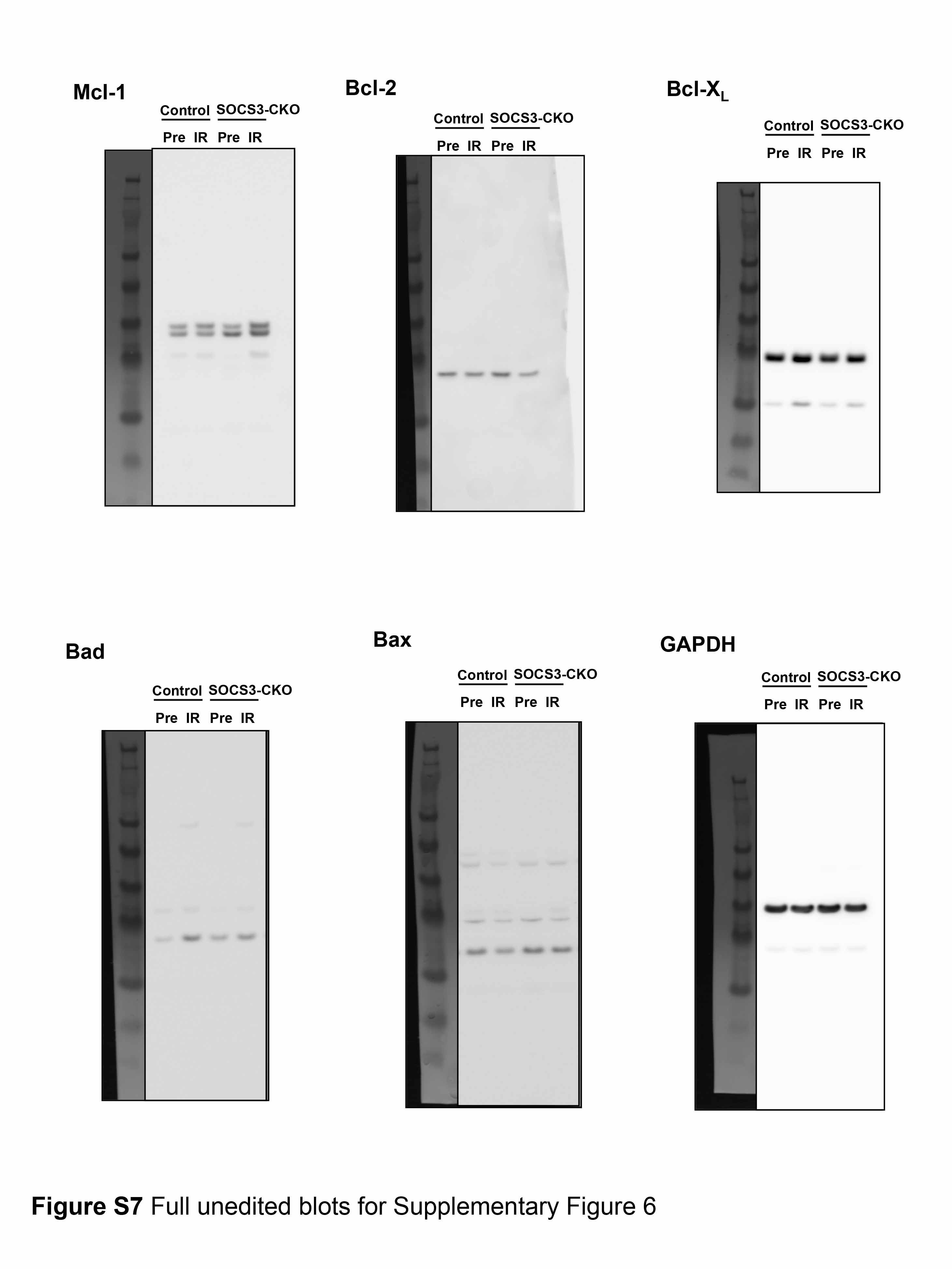

Supplement: S7 Fig — Blots were probed using antibodies against myeloid cell leukemia-1 (Mcl-1), Bcl-xL, Bcl-2, Bad, Bax, and GAPDH. (JPG) [file pone.0127942.s007.jpg]
